# Structure-based development of 3,5-dihydroxybenzoyl-hydrazineylidene as tyrosinase inhibitor; in vitro and in silico study

**DOI:** 10.1038/s41598-024-52022-6

**Published:** 2024-01-17

**Authors:** Azzam Bagheri, Shahram Moradi, Aida Iraji, Mohammad Mahdavi

**Affiliations:** 1 Faculty of Chemistry, Islamic Azad University, North Tehran Branch, Tehran, Iran; 2 Research Center for Traditional Medicine and History of Medicine, Department of Persian Medicine, School of Medicine, Shiraz University of Medical Sciences, Shiraz, Iran; 3 Stem Cells Technology Research Center, Shiraz University of Medical Sciences, Shiraz, Iran; 4 Central Research Laboratory, Shiraz University of Medical Sciences, Shiraz, Iran; 5 Endocrinology and Metabolism Research Center, Endocrinology and Metabolism Clinical Sciences Institute, Tehran University of Medical Sciences, Tehran, Iran

**Keywords:** Chemical biology, Drug discovery

## Abstract

A series of new analogs of 3,5-dihydroxybenzoyl-hydrazineylidene conjugated to different methoxyphenyl triazole (**11a-n**) synthesized using click reaction. The structures of all synthesized compounds were characterized by FTIR, ^1^H, ^13^C-NMR spectroscopy, and CHO analysis. The tyrosinase inhibitory potential of the synthesized compounds was studied. The newly synthesized scaffolds were found to illustrate the variable degree of the inhibitory profile, and the most potent analog of this series was that one bearing 4-methoxyphenyl moiety, and exhibited an IC_50_ value of 55.39 ± 4.93 µM. The kinetic study of the most potent derivative reveals a competitive mode of inhibition. Next, molecular docking studies were performed to understand the potent inhibitor's binding mode within the enzyme's binding site. Molecular dynamics simulations were accomplished to further investigate the orientation and binding interaction over time and the stability of the **11m**-tyrosinase complex.

## Introduction

Tyrosinase (EC 1.14.18.1) is a type-3 copper-containing metalloenzyme present in plants, fungi, bacteria, and mammals. Tyrosinase is a glycoprotein located in the membrane of the melanosome, a vesicle inside the melanocyte^1^. Tyrosinase is a key enzyme that catalyzes critical steps in melanin biosynthesis, including hydroxylation of L-tyrosine to 3,4-dihydroxyphenylalanine (L-DOPA), oxidation of L-DOPA to DOPAquinone. Also, it was reported that tyrosinase participates in the oxidation of 5,6-dihydroxyindole to indolequinone. The final products of the melanogenesis process are eumelanin (mostly a dark brown to black polymer) and pheomelanin (a yellow to red polymer)^2^ resulting in the formation of macromolecular pigments, melanin.

Abnormal pigment levels are linked to several problems, including pigmented patches, skin hyperpigmentation, postinflammatory hyperpigmentation, maturational dyschromia, periorbital hyperpigmentation, melasma, and Riehl melanosis^3^. Also, some evidence exists about the correlation between neuromelanin and CNS disease. On the other hand, the undesirable enzymatic browning of vegetables and fruit related to melanin synthesis is detrimental to the quality and color of the products^4^. Regarding the above considerations and the key role of tyrosine in melanogenesis, the discovery of new tyrosinase inhibitors is highly needed. Over time, different natural and synthetic tyrosinase inhibitors have been introduced. Currently, tyrosinase inhibitors, including kojic acid, arbutin, azelaic acid, and hydroquinone, possess undesirable side effects such as low clinical efficacy and carcinogenicity. Therefore, synthesizing novel inhibitors for medical and cosmetic applications is of great interest^5^.

**Figure 1:**
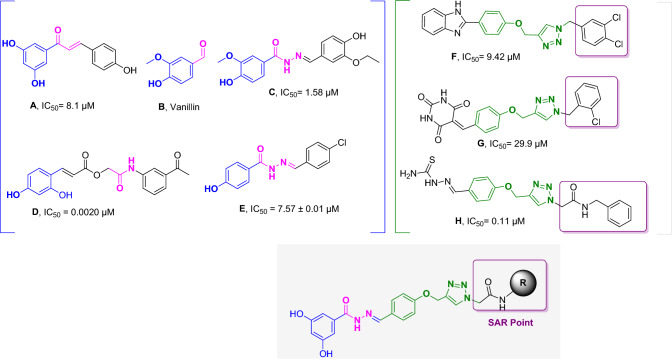
Potent inhibitors of mushroom tyrosinase from previous studies and newly designed compound.

Chalcone has been introduced as a potent tyrosinase inhibitor through screening a library of compounds and different study evaluations. Vaya et al. examined non-, mono-, di-, tri-, or tetra-substituted hydroxyl derivatives as tyrosinase inhibitors, and amongst compound **A** exhibited the best potency^6^. Vanillin (Compound **B**), a natural flavoring agent, exhibited good tyrosinase inhibition, and vaniline–benzylidenehydrazine (Compound **C**) derivatives were developed^7^. The most potent compound in this series is exhibited in Fig. 1. The molecular docking study showed that the OH groups participate in critical interactions with two copper cofactors, plus His85 and His263, essential for tyrosinase inhibition. Also, compound **D**^8^ bearing OH moiety on the phenyl exhibited significant inhibition against tyrosinase. Compound **E** disclosed an IC_50_ near the positive control and Cu^2+^ chelation potential with a mole fraction of 1:2 stoichiometry^9^.

Different studies also confirmed the high potency of triazole and its analogs as anticancer^10–12^, anti-microbial^13,14^, anti-inflammatory^15,16^, and anti-melanogenic agents as tyrosinase inhibitors. In this category, triazole-benzoimidazole (Compound **F)** with IC_50_ = 9.42 μM is a good example^17^. A new series of new triazole derivatives (Compound **G**) was reported, and it was shown that moieties with the ability to form hydrogen bonds with His85 and His244 improve the potency^18^. Recently, phenoxy methyl triazole conjugated with thiosemicarbazide (Compound **H**) was reported to induce the tyrosinase inhibitory effects with IC_50_ values of 0.11 μM and 0.17 μM in the presence of L-tyrosine and L-DOPA as substrates. The proposed mechanism of the high potency of tyrosinase inhibitor was related to its Cu chelatory potential. Compound **H** significantly reduced the melanin content in skin melanoma cells to 39.8% at 8 µM. The main interactions with tyrosinase active site were seen between the phenoxy group and His263 and Ala286. Residue Val283 formed an H-bond interaction with the triazole ring and two pi-sigma interactions with triazole and methoxybenzene rings^19^. As a result, phenoxy-triazole was chosen as a valuable starting point.

With these results in hand, 3,5-dihydroxybenzohydrazide was chosen that has similarity with native substrate L-tyrosine to explore the structural requirements of tyrosinase-inhibitory activity. The presence of such OH moiety provides the minimal structural requirements of tyrosinase inhibition. On the other hand, aryl methoxy-triazole scaffolds were connected to 3,5-dihydroxybenzohydrazide moiety through Schiff base reaction, developing and extending the structure–activity relationships (SARs), and different derivitization were conducted at the R position. It was proposed that aryl methoxy-triazole not only provides the optimum bulkiness to occupy the pocket of the enzyme but also might interact with the critical residue of the binding site to hinder the oxidation process. The designed compounds were synthesized and evaluated as possible tyrosinase inhibitors. Next, the most potent derivative was subjected to the kinetic study to determine the type of inhibition. Furthermore, molecular docking and molecular dynamic simulations were also performed.

## Results and discussion

### Synthesis

**Figure 2:**
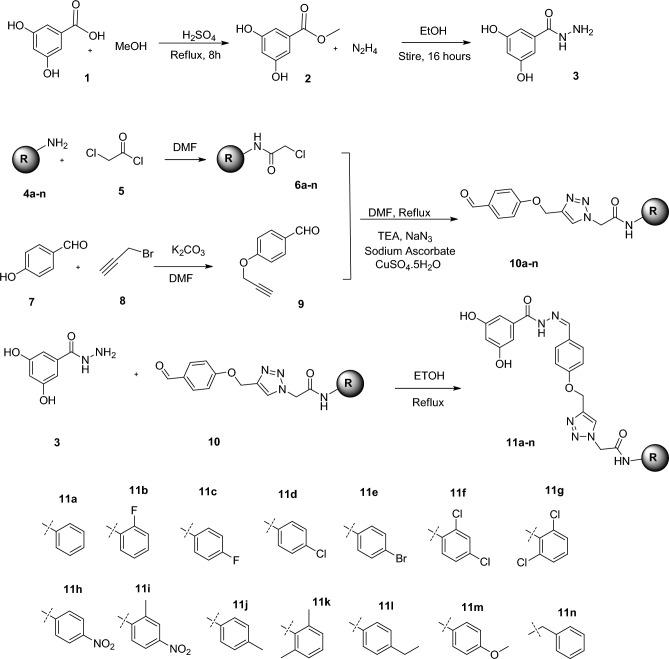
Synthesis of compounds **11a-n**.

Synthesis of the target compounds **11a-n** was schematically described in Fig. 2. Briefly, 3,5-dihydroxybenzoic acid (compound **1**) was allowed to react with methanol under the refluxed condition to conduct an esterification reaction. After 8 h, the methanol was evaporated to obtain methyl 3,5-dihydroxybenzoate (compound **2**). This methyl ester was then reacted with hydrazine hydrate to produce the intermediate 3,5-dihydroxybenzoic acid hydrazide (compound **3**). In a separate reaction, chloroacetyl chloride (compound **5**) was added to aniline derivatives (compound **4a-n**) in DMF, yielding compound **6a-n**. In another reaction vessel, propargyl bromide (compound **8**) was introduced to 4-hydroxybenzaldehyde (compound **7**) and potassium carbonate to produce *O*-propargyl benzaldehyde (compound **9**). Additionally, aryl acetamide derivatives were refluxed with sodium azide and triethylamine (TEA), followed by the addition of compound **9** and catalytic CuSO_4_.5H_2_O and sodium ascorbate to produce the **10a-n**. Finally, a mixture of compounds **10a-n** and 3,5-dihydroxybenzohydrazide in the presence of acetic acid was refluxed in ethanol, followed by purification through diethyl ether crystallization to obtain the desired products, **11a-n**^20,21^.

### In vitro inhibition of tyrosinase

Inhibitory activities against tyrosinase were determined in the colorimetric assay for all compounds. The results are shown in Table 1. 3,5-Dihydroxybenzoyl-hydrazineylidene compounds were synthesized to assess the importance of different substituents at R positions of the terminal aryl ring.

**Table 1:** Tyrosinase inhibitory activities of **11a-n**.


Compound	R	% inhibition at 100 µM^a^	IC_50_ (µM) ± RSD^b^
**11a**	Phenyl	53.87 ± 1.53	90.53 ± 1.98
**11b**	2-fluorophenyl	22.99 ± 1.20	–
**11c**	4-fluorophenyl	32.28 ± 2.76	–
**11d**	4-chlorophenyl	22.82 ± 0.53	–
**11e**	4-bromophenyl	14.13 ± 2.31	–
**11f**	2,4-dichlorophenyl	19.54 ± 2.48	–
**11g**	2,6-dichlorophenyl	27.87 ± 1.83	–
**11h**	4-nitrophenyl	22.74 ± 2.86	–
**11i**	2-methyl-4-nitrophenyl	15.05 ± 2.29	–
**11j**	4-methylphenyl	15.22 ± 4.33	–
**11k**	2,6-dimethylphenyl	38.59 ± 0.74	–
**11l**	4-ethylphenyl	55.52 ± 4.63	87.54 ± 7.99
**11m**	4-methoxyphenyl	78.81 ± 7.35	55.39 ± 4.93
**11n**	Benzyl	17.48 ± 1.79	–
**Kojic acid**^**c**^		23.64 ± 2.56	

^a^Solubility < 100 µM.

^b^50% inhibitory concentration (IC_50_).

^c^Kojic acid as the positive control.

Compound **11a**, serving as the primary unsubstantiated derivative, displayed an IC_50_ value of 90.53 µM with a modest 53.87% inhibition observed at 100 µM concentration. This initial finding prompted us to investigate the impact of electron-withdrawing groups on the compound's potency, leading to the design and synthesis of compounds **11b-h**.

Incorporating a 2-fluorophenyl group in compound **11b**, reduced the inhibitory potency, with only 22.99% inhibition at 100 µM. This result suggests that the electron-withdrawing nature of the fluorine atom has a detrimental effect on the compound's activity. While shifting the substituent from the *ortho* to *para* position in compound **11c** enhanced the inhibitory activity, with a measured inhibition of 32.28% at 100 µM. This suggests that the *para* position is more favorable for improving the compound's potency.

A more detailed evaluation of *para*-position substitutions revealed that 4-fluorophenyl (**11c**) had the highest potency, surpassing 4-chlorophenyl (**11d**) > 4-bromophenyl (**11e**). This ranking indicates that increasing the bulkiness of the *para*-position substituents negatively correlates with the inhibitory potency. The observed trend underscores the importance of understanding the impact of both electron-withdrawing and steric effects in optimizing the compound's activity.

Furthermore, introducing multi-halogen substitutions at different positions of the phenyl ring was attempted to improve inhibition potentially. However, it was observed that no improvement in potency was achieved in compounds **11f** (R = 2,4-dichlorophenyl) and **11g** (R = 2,6-dichlorophenyl) when compared to the unsubstituted analog **11a**.

Given that the halogen-substituted group did not significantly improve the potency compared to the unsubstituted moiety, introducing nitro groups was considered an alternative approach to assess their electron-donating potential and the potential for hydrogen-bond interactions. It was observed that compounds **11h** (R = 4-nitrophenyl) and **11i** (R = 2-methyl-4-nitrophenyl), as nitro-substituted derivatives, exhibited lower inhibitory potencies when compared to **11a** as the unsubstituted derivative.

In light of the lack of activity observed in the electron-withdrawing substituted derivative, the synthesis of electron-donating groups was pursued. Introducing a *para*-methyl group resulted in a significant activity loss, with only 15.22% inhibition observed at 100 µM. However, the inhibitory activity of compound **11k**, which featured 2,6-dimethyl substitutions, improved when compared to the monomethyl substituted group, with an inhibition rate of 38.59% at 100 µM. Additionally, introducing a larger electron-donating group like 4-ethyl substitution (**11l**) further enhanced the compound's potency, resulting in an IC_50_ value of 87.54 µM. This data underscores the importance of considering the electron-donating nature and steric effects when optimizing the inhibitory potential of these derivatives.

Remarkably, the *para*-methoxy group emerged as the most effective substitution, with compound **11m** displaying the highest potency, featuring an IC_50_ value of 58.88 μM. This finding underscores the potential of electron-donating properties, particularly when coupled with heteroatoms, to facilitate hydrogen-bond interactions, positively influencing the compound's inhibitory activity.

Additionally, introducing a benzyl group (**11n**), which elongates the linker, resulting in an inhibition of 17.48% at 100 µM. However, it is apparent that extending the linker is not well-tolerated and may not be conducive to improving the compound's inhibitory potency.

### Enzyme inhibitory kinetics

**Figure 3:**
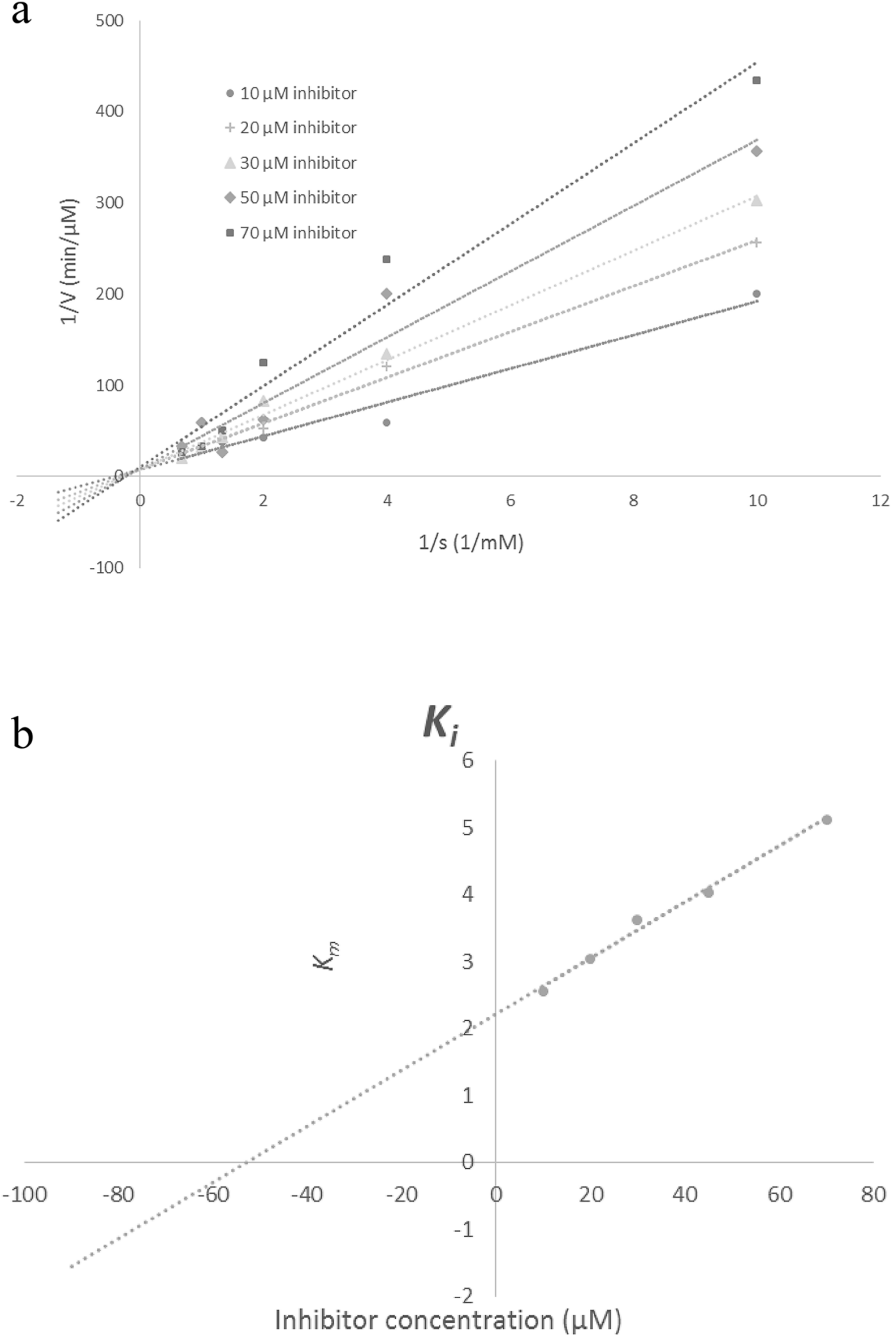
**(a)** Line weaver-burk plot of **11m** against tyrosinase; (**b**) The secondary plot between the *K*_*m*_ and various concentrations of **11m**.

The enzyme inhibitory interaction mechanism of **11m** with the binding site of tyrosinase was determined using Michaelis–Menten kinetic studies. The inhibitor exhibits a dose-dependent inhibition of the enzyme tyrosinase. Inhibition kinetics was analyzed by the Lineweaver–Burk plot with 1/V_max_*vs*. 1/[S] at different doses of **11m** (Fig. 3a). Results exhibited that the Michaelis–Menten constant (*K*_*m*_) changes while that of 1/V_max_ remained the same, representing the competitive nature of the most potent inhibitor (**11m**) (Fig. 3a). The dissociation constant *K*_*i*_ for **11m** was 52.77 μM as shown in Fig. 3b.

### Docking study

The molecular docking study was conducted to provide insights into the binding mode of **11m**, the most potent derivative against the tyrosinase enzyme.

The validation of the molecular docking process was initially conducted through the redocking of a co-crystallized ligand, tropolone, within the binding site of the enzyme. The procedure resulted in an RMSD (root mean square deviation) value of less than 2 Å, confirming that the docking approach was reliable. This low RMSD value implies a strong correlation between the docked conformation and the crystalographic conformation, suggesting that the docking protocol accurately predicts the ligand's orientation and position within the enzymatic binding site. This validation step is critical, as it establishes the credibility of the docking method for further exploration and analysis of potential ligand-enzyme interactions in subsequent studies.

The in silico studies conducted on the designed analogs were examined. As determined by the molecular docking analysis results presented in Table 2, the docking scores of the derivatives against tyrosinase ranged from – 7.570 to – 4.157 kcal/mol. Notably, there was a positive correlation between these docking scores and the biological results, strengthening the validity of the approach.

**Table 2:** Docking scores resulted of **11a–n** against tyrosinase.

Compound	R	Type of interactions	Residue	moiety
**11a**	– 5.386	H-bound	Thr261	NH of acetamide
		H-bound	Glu256	OH of 2,4-dihydroxyphenyl
		Pi-pi stacking	Trp227	Benzyl
		Pi-pi stacking	His85	2,4-dihydroxyphenyl
		Pi-pi stacking	His259	2,4-dihydroxyphenyl
**11b**	– 5.082	H-bound	Asn260	OH of 2,4-dihydroxyphenyl
		H-bound	Glu256	OH of 2,4-dihydroxyphenyl
		Pi-pi stacking	His85	2,4-dihydroxyphenyl
		H-bound	Ser282	Triazole
**11c**	– 5.002	H-bound	Arg268	C = O of acetamide
		Pi-pi stacking	His259	2,4-dihydroxyphenyl
		Pi-pi stacking	His261	2,4-dihydroxyphenyl
**11d**	– 5.229	H-bound	Asn260	OH of 2,4-dihydroxyphenyl
		H-bound	Glu256	OH of 2,4-dihydroxyphenyl
		Pi-pi stacking	His85	2,4-dihydroxyphenyl
		H-bound	Ser282	Triazole
**11e**	– 4.351	H-bound	Glu256	OH of 2,4-dihydroxyphenyl
		Pi-pi stacking	His85	2,4-dihydroxyphenyl
**11f**	– 4.905	H-bound	Glu256	OH of 2,4-dihydroxyphenyl
		Pi-pi stacking	His85	2,4-dihydroxyphenyl
		Pi-pi stacking	His259	2,4-dihydroxyphenyl
**11g**	– 5.825	H-bound	Arg268	C = O of acetamide
		H-bound	Arg268	Triazole
		Pi-pi stacking	His85	2,4-dihydroxyphenyl
		Pi-pi stacking	His263	2,4-dihydroxyphenyl
**11h**	– 5.751	H-bound	Ser282	OH of 2,4-dihydroxyphenyl
		H-bound	Asn260	C = O of hydrazineylidene
		Pi-pi stacking	His85	2,4-dihydroxyphenyl
		Pi-cation	Trp227	4-NO_2_ phenyl
**11i**	– 4.157	H-bound	Ser282	OH of 2,4-dihydroxyphenyl
		Pi-pi stacking	His263	2,4-dihydroxyphenyl
		Pi-cation	Trp227	4-NO_2_ phenyl
**11j**	– 4.416	H-bound	Glu256	OH of 2,4-dihydroxyphenyl
		Pi-pi stacking	His85	2,4-dihydroxyphenyl
		Pi-pi stacking	His259	2,4-dihydroxyphenyl
**11k**	– 6.276	H-bound	Val283	OH of 2,4-dihydroxyphenyl
		H-bound	Asn260	C = O of hydrazineylidene
		Pi-pi stacking	His263	2,4-dihydroxyphenyl
		Metal- coordination	Cu400	OH of 2,4-dihydroxyphenyl
		Metal- coordination	Cu401	OH of 2,4-dihydroxyphenyl
**11l**	– 6.254	H-bound	Asn260	C = O of hydrazineylidene
		H-bound	Arg268	C = O of acetamide
		Pi-pi stacking	His259	2,4-dihydroxyphenyl
		Pi-pi stacking	His263	2,4-dihydroxyphenyl
		Pi-cation	Arg268	Triazole
		Metal- coordination	Cu400	OH of 2,4-dihydroxyphenyl
**11m**	– 8.082	H-bound	Asn81	OCH_3_
		H-bound	His85	C = O of acetamide
		Pi-pi stacking	His263	Phenoxy
		Pi-pi stacking	His85	2,4-dihydroxyphenyl
		Metal- coordination	Cu400	C = O of hydrazineylidene
		Metal- coordination	Cu400	NH of hydrazineylidene
		Metal- coordination	Cu401	N of hydrazineylidene
		H-bound	Glu98	OH of 2,4-dihydroxyphenyl
		Pi-pi stacking	His61	2,4-dihydroxyphenyl
		Pi-pi stacking	Tyr97	2,4-dihydroxyphenyl
		Pi-pi stacking	Phe292	2,4-dihydroxyphenyl
**11n**	– 4.675	H-bound	Ser282	OH of 2,4-dihydroxyphenyl
		H-bound	Asn260	C = O of hydrazineylidene
		Pi-pi stacking	His85	2,4-dihydroxyphenyl
		Pi-pi stacking	His264	Triazole

Particularly, compounds **11m** and **11l** were identified as the most potent inhibitors. Compound **11m**, with an IC_50_ value of 55.39 ± 4.93 µM, demonstrated a remarkable docking score of – 7.57 kcal/mol. In contrast, compound **11l**, exhibiting an IC_50_ value of 87.54 ± 7.99 µM, was associated with a docking score of – 6.254 kcal/mol. These findings underscore their prominence as active inhibitors in the series. Conversely, the compounds considered as less active, including **11i**, **11j**, **11e**, and **11f**, were noted for their relatively minimal docking scores against tyrosinase, recorded – 4.157, – 4.416, – 4.351, and – 4.905 kcal/mol, respectively. This denotes a clear separation between active and less active molecules regarding their potency.

Further examination into the binding interactions has revealed that the most potent inhibitors in our dataset primarily interact with the Cu^+2^ cofactor through a metal coordination bond. This type of interaction is critical for inhibiting tyrosinase activity, suggesting that the metal binding is a key pathway for inhibitory action amongst the active compounds studied.

**Figure 4:**
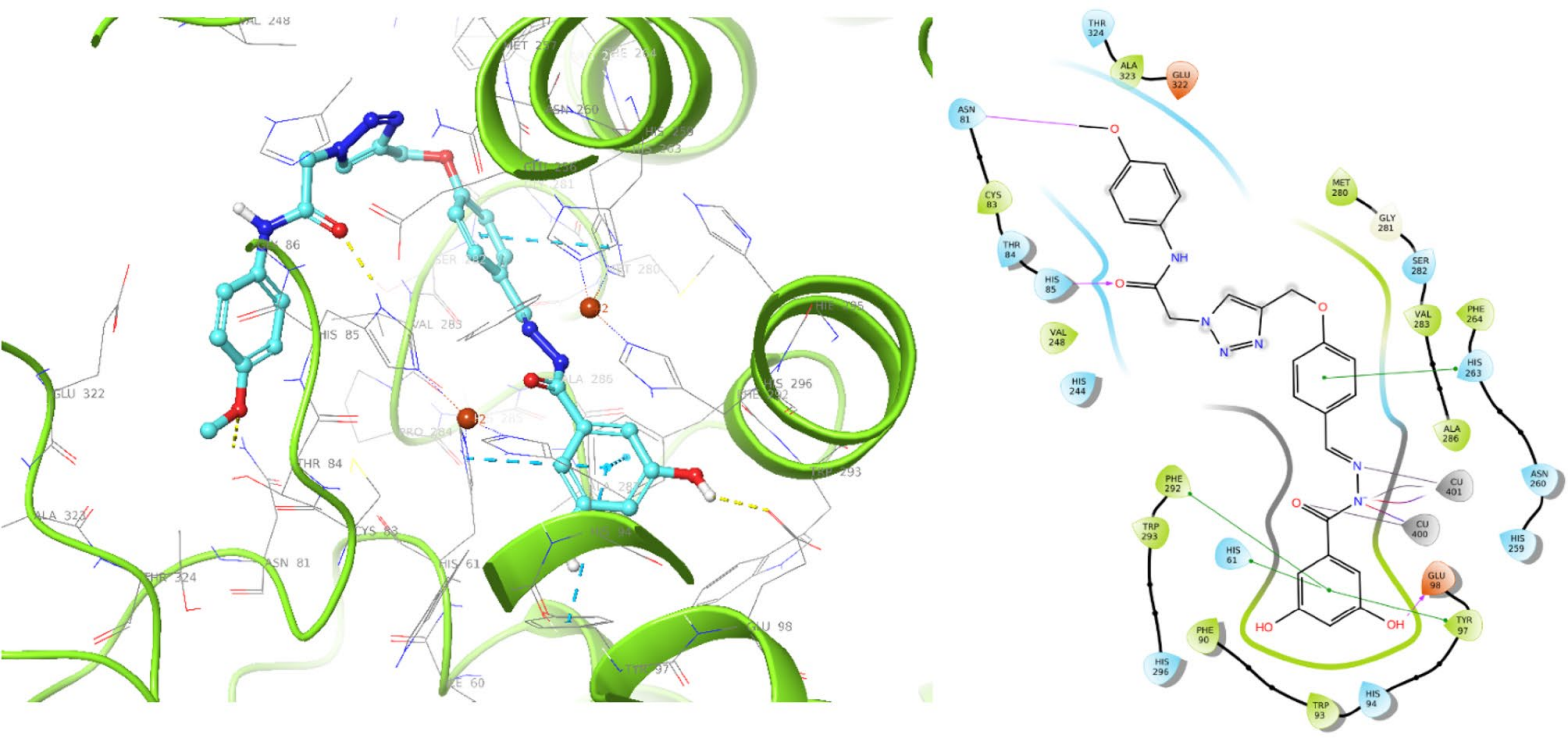
Representation of compound **11m** within the active site of tyrosinase.

The docking results of **11m** as the most potent compound, characterized by its chalcone structure, displayed five noteworthy interactions. These interactions encompassed associations with both Cu^2+^ ions, H-bond interactions with Glu98, and three π-π stacking interactions with His61, Tyr97, and Phe292.On the other side of the molecule, the phenolic linker exhibited another π-π stacking interaction with His263, and the amide group formed an H-bond with the crucial His85, along with another H-bond with Asn81. These results confirm that **11m** interacts critically with Cu ions and His residues (Fig. 4).

### Molecular dynamics simulation

**Figure 5:**
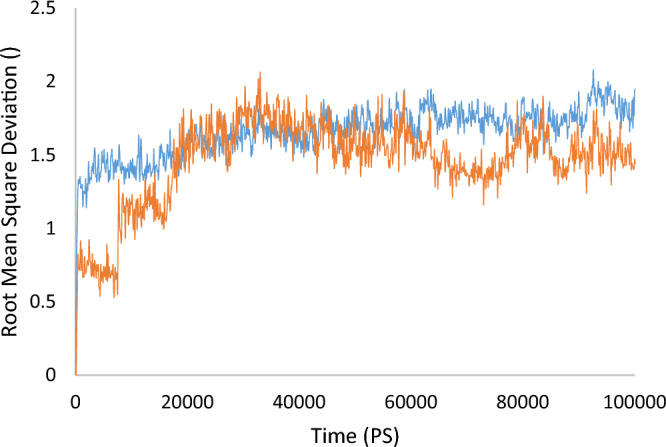
RMSD plot of the tyrosinase in complex with compound **11m** in the MD simulation time. RMSD values of the apo-enzyme are depicted in blue, and the **11m**-enzyme is exhibited in orange.

The molecular dynamics (MD) simulation was performed to confirm the **11m** stability over the enzyme active site. First, the root mean square deviation (RMSD) of the enzyme's backbone was analyzed over during 100 ns MD simulation to study the perturbation of the protein–ligand complex. As shown in Fig. 5, atoms of the protein alone fluctuated at around 1.75 Å. Specifically, within the first 8 ns of the simulation, the RMSD experienced a pronounced increase. Subsequently, between 8–18 ns, it stabilized at an RMSD value of 1.75 Å. Notably, for the remaining duration of the simulation, the complex exhibited a consistently lower RMSD compared to the protein alone, which maintained an RMSD of around 1.4 Å, confirming the stability of the complex.

**Figure 6:**
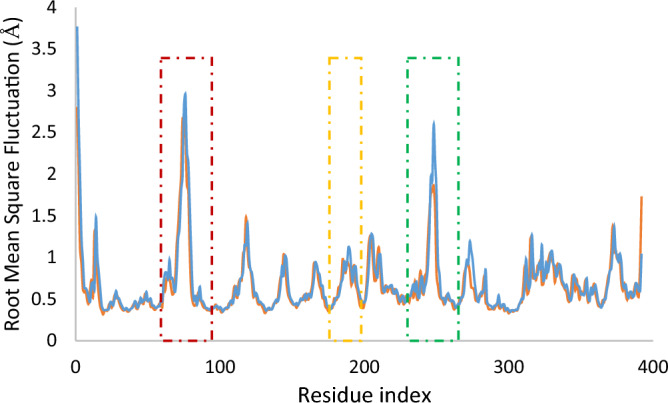
RMSF plot of the tyrosinase residues in complexed with **11m**. RMSF values of the apo-enzyme are depicted in blue, and the **11m**-enzyme is exhibited in orange.

Next, The root mean square fluctuation (RMSF) is also evaluated. RMSF recorded the local changes along the protein chain during the MD run. As exhibited, the secondary structure elements like α-helical and β-strand are usually rigid. The high fluctuations observed in the RMSD values can be attributed to the unstructured region of the enzyme (Fig. 6). As indicated, the reduction in movement in the region spanning residues 68–81 (the red dashed line), residues 245 to 251 (the yellow dashed line), along with residues 187–195 (the green dashed line), compared to the apoenzyme in these areas, played a significant role in stabilizing the complex.

**Figure 7:**
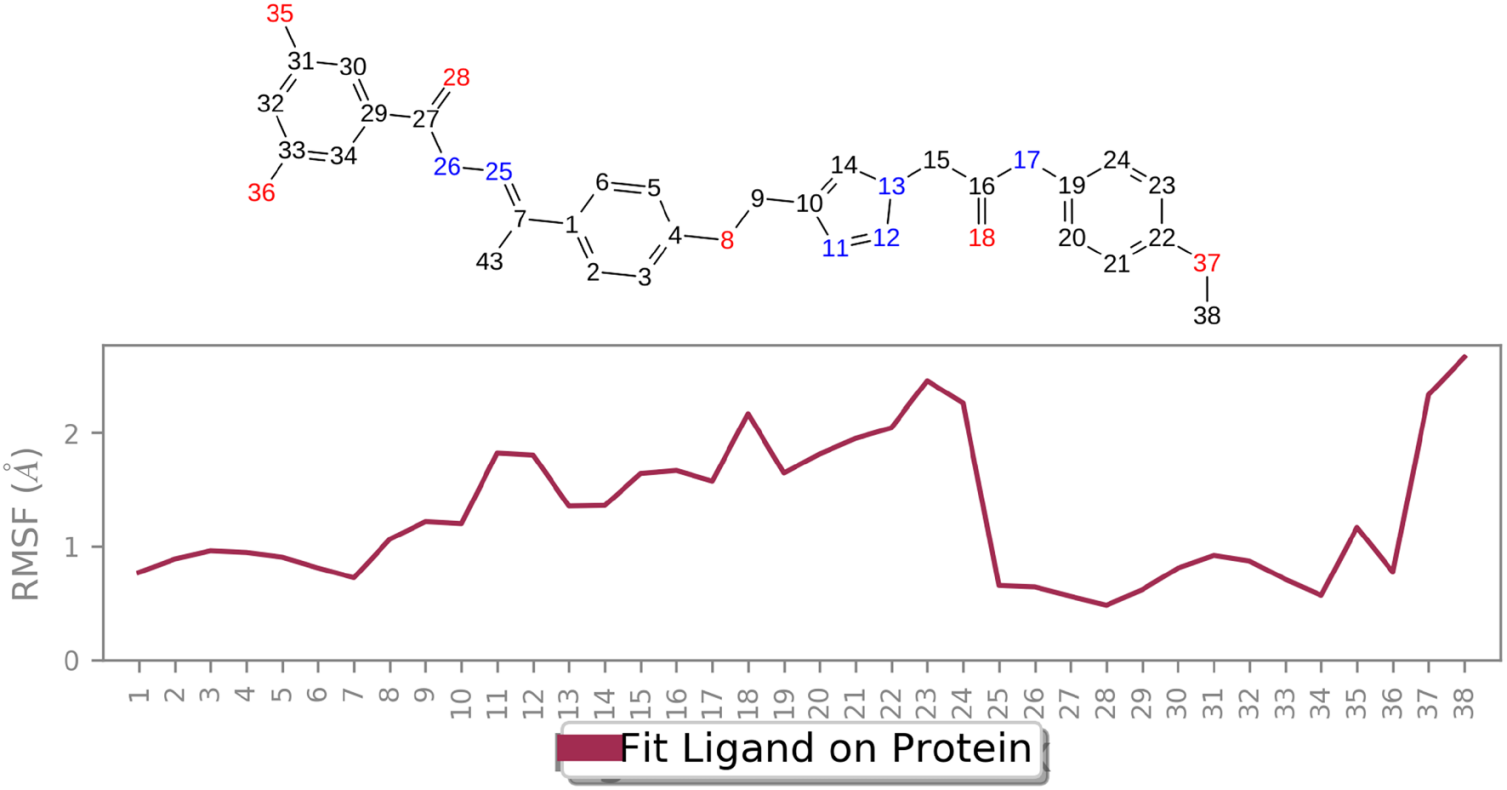
L-RMSF graph of the heavy atoms of **11m** in complex with tyrosinase.

In Fig. 7 depicts the Ligand Root Mean Square Fluctuation (L-RMSF) values of the heavy atoms of ligand 11m when bound to tyrosinase. With the exception of the 4-methoxyphenyl regions, all atoms in **11m** exhibit RMSF values below 2 Å. This minimal fluctuation signifies a stable complex formation with tyrosinase, primarily due to robust intermolecular interactions that restrict their mobility during the molecular dynamics simulation. This persistent binding interaction strongly suggests that ligand **11m** holds promising potential as an effective tyrosinase inhibitor.

**Figure 8:**
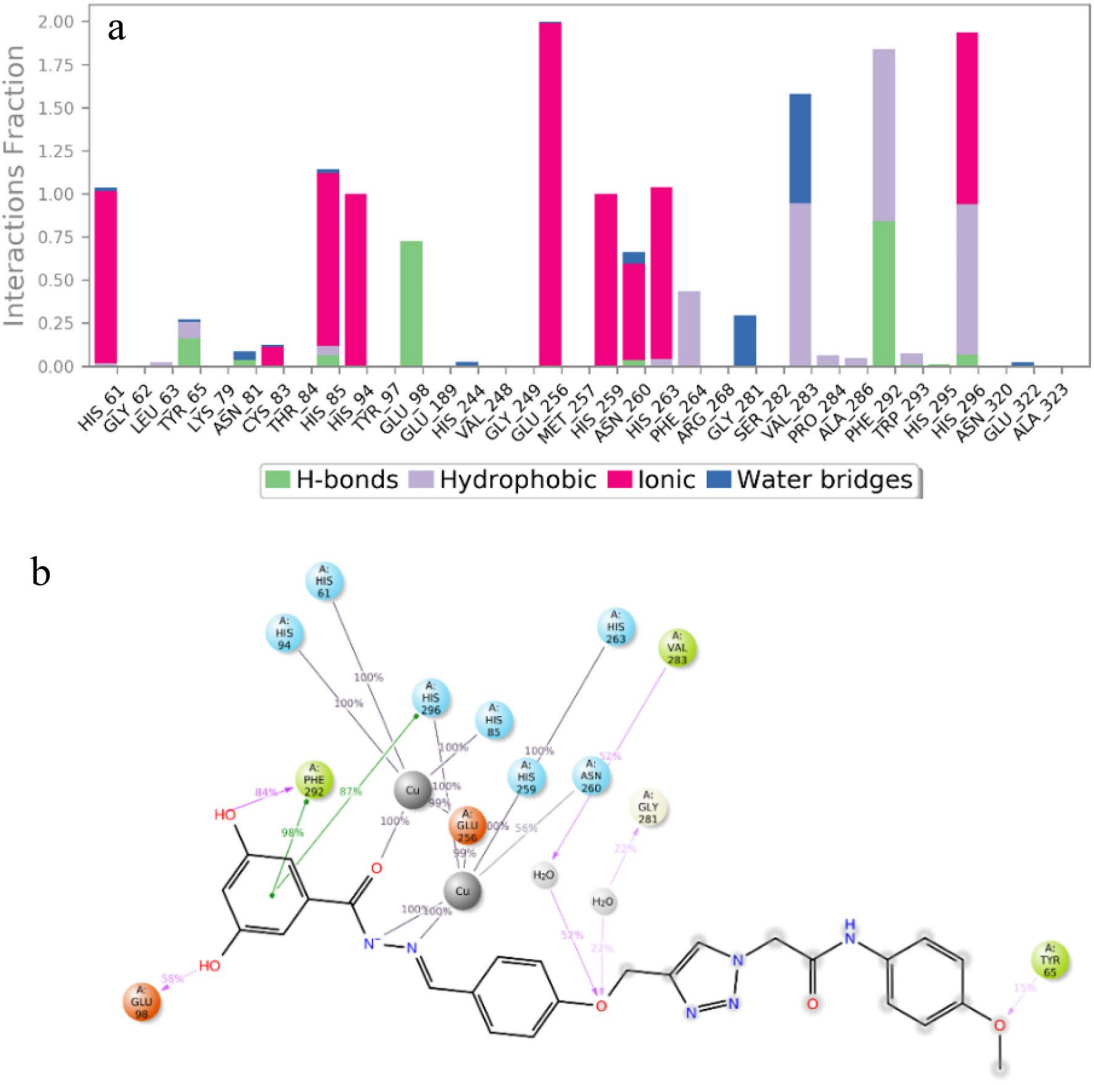
(**a**) Protein interaction types with the **11m** throughout the simulation, (**b**) 2D ligand interactions with the protein residues.

Interaction types with the ligand throughout the simulation run are exhibited in Fig. 8a, and the interaction with each residue is exhibited in Fig. 8b. 3,5-dihydroxybenzoyl displayed multiple interactions within the system. It engaged in two hydrogen-bond interactions with Glu98 and Phe292 and two pi-pi stacking interactions with Phe292 and His296. Notably, the C=O group of the benzoylhydrazone moiety consistently participated in metal coordination with the copper cofactor throughout the entire simulation period (100%). Moreover, the hydrazine group formed an essential metal interaction with another copper ion involved in the oxidation process. This hydrazine linker was observed to effectively engage with the critical copper cofactor throughout the total simulation time (100%), ultimately impeding the oxidation process in the melanogenesis pathway. Within the molecular structure, the phenoxy linker in the middle of the molecule demonstrated a hydrogen bond interaction with Val283, facilitated by water molecules, occurring during 52% of the MD simulation run. This phenoxy linker recorded another H-bound interaction with Asn260 mediated with water (52%). Additionally, the 4-methoxyphenyl group at the terminal of the molecule exhibited a hydrogen bond interaction with Tyr65. These critical interactions confirm the high potency of **11m**, which properly occupies the binding site.

In addition to the interaction analysis, the Prime/MM-GBSA module was used to estimate the strengths of interactions between the ligand–protein complex generated by the clustering method. ΔG_bind_ of tyrosinase/compound **11m** complex was estimated to be – 43.46 kcal/mol.

### ADMET properties and in silico toxicity

Table 3 demonstrates drug-likeness prediction for **11a-n**. Drug-likeness refers to the set of properties that make a molecule suitable for use as a drug. Lipinski's Rule of Five evaluates a compound's drug-likeness based on its physicochemical properties. This rule includes the molecular weight (MW ≤ 500), the number of hydrogen bonding acceptors (≤ 10), the number of hydrogen bonding acceptors (≤ 5), the lipophilicity index (logP ≤ 5), rotatable bond count (≤ 10) and polar surface area (≤ 140) of compounds^22^ . Regardless of molecular weight, most compounds have successfully met Lipinski's rule^23^.

**Table 3:** Drug-likeness prediction for **11a-n**.

Compd	Molecular weight	Log P	Rotatable bonds	H-bond acceptors	H-bond donors	Surface area
11a	486.48	2.67	9	9	4	205.21
11b	504.47	2.81	9	9	4	209.37
11c	504.47	2.81	9	9	4	209.37
11d	520.93	3.32	9	9	4	215.51
11e	565.38	3.43	9	9	4	219.08
11f.	555.37	3.97	9	9	4	225.82
11g	555.37	3.97	9	9	4	225.82
11h	531.48	2.57	10	11	4	219.86
11i	545.51	2.88	10	11	4	226.23
11j	500.51	2.97	9	9	4	211.57
11k	514.54	3.28	9	9	4	217.94
11l	514.54	3.23	10	9	4	217.94
11m	516.51	2.67	10	10	4	216.69
11n	500.51	2.34	10	9	4	211.57

ADMET stands for Absorption, Distribution, Metabolism, Excretion, and Toxicity of pharmaceutical compounds within a biological system. These properties are crucial component of drug development that can significantly improve the safety, efficacy, and efficiency of bringing new medications. Table 4 presents the ADMET predictions for all compounds, calculated using the pkCSM online servers^23^. The estimated Human Intestinal Absorption (HIA) of the compounds ranged from 60.123 to 67.123, indicating a relatively high absorption rate within the gastrointestinal tract. This suggests that the compounds have favorable pharmacokinetic profiles for oral administration, an essential characteristic for effective systemic drug delivery. The compounds under investigation are estimated to have a low steady-state volume of distribution, ranging from – 2.735 to – 0.386 log L/kg, which indicates limited distribution primarily to the plasma and extracellular spaces rather than extensive tissue penetration. This profile potentially minimizes adverse effects associated with tissue accumulation, making these compounds particularly suitable for treatments necessitating rapid systemic availability and clear dose–response relationships. Regarding metabolism, all compounds were predicted not to be inhibitors of CYP2D6 and CYP2C19 while expected to be CYP3A4 and CYP2C9 inhibitors. This profile may exhibit an advantage by minimizing the risk of adverse drug interactions mediated via CYP2D6 and CYP2C19 enzymes involved in the metabolism of a wide range of pharmaceuticals. The total clearance of derivatives is in the range of 0.267 to – 0.437. A higher clearance rate may be desired to achieve a rapid onset of action and shorter duration. In contrast, slower clearance may be necessary to maintain therapeutic levels over an extended period. In silico models have quantified the maximum tolerated dose for humans between 0.270 and 0.319 mg/kg/day for the compounds studied, denoting a closely defined dosing range advantageous for further refinement of dosage regimens in clinical trials.

**Table 4:** ADMET prediction of the synthesized compounds.

Compd	Absorption	Distribution^b^		Metabolism^b^				Excretion^b^	Toxicity^b^		
	HIA%	Caco2 permeability (log Papp in 10^–6^ cm/s)	VDss (log L/Kg)	CYP3A4 inhibition	CYP2D6 inhibition	CYP2C9 inhibition	CYP2C19 inhibition	Total Clearance (log ml/min/kg)	Max. tolerated dose human (Log mg/kg/day)	Oral Rat Acute Toxicity LD_50_ (mol/kg)	hERG1 inhibitor
**11a**	63.07	0.388	– 0.559	Yes	No	Yes	No	0.101	0.311	2.424	No
**11b**	67.123	0.347	– 0.599	Yes	No	Yes	No	– 0.055	0.319	2.419	No
**11c**	67.123	0.347	– 0.599	Yes	No	Yes	No	– 0.055	0.319	2.419	No
**11d**	64.24	0.368	– 0.565	Yes	No	Yes	No	– 0.416	0.317	2.428	No
**11e**	63.982	0.362	– 0.559	Yes	No	Yes	No	– 0.437	0.317	2.43	No
**11f.**	65.422	0.348	– 0.569	Yes	No	Yes	No	– 0.223	0.324	2.433	No
**11g**	63.070	0.388	– 0.559	Yes	No	Yes	No	0.101	0.311	2.424	No
**11h**	60.123	0.195	– 1.055	Yes	No	Yes	No	0.126	0.271	2.52	No
**11i**	60.591	0.195	– 1.039	Yes	No	Yes	No	0.142	0.270	2.521	No
**11j**	63.538	0.388	– 0.539	Yes	No	Yes	No	0.105	0.308	2.43	No
**11k**	64.006	0.389	– 2.735	Yes	No	Yes	No	0.119	0.305	2.436	No
**11l**	63.794	0.31	– 0.729	Yes	No	Yes	No	0.139	0.282	2.487	No
**11m**	65.211	0.356	– 2.735	Yes	No	Yes	No	0.062	0.318	2.428	No
**11n**	60.417	0.394	– 0.386	Yes	No	Yes	No	0.267	0.238	2.415	No

^a^HIA (Human Intestinal Absorption): > 80% is high and < 30% is poor; VDss (steady-state volume of distribution): log L/Kg: > 0.45 is high and < – 0.15 is low. Caco-2 permeability > 0.90 considered.

Meanwhile, the oral rat acute toxicity studies showed a narrow range from 2.415 to 2.487, suggesting a consistent toxicity profile in this model, which rationalizes the extrapolation to potential human toxicology outcome. hERG1 inhibition is a valuable parameter in ADME-T prediction, particularly for assessing the risk of drug-induced long QT syndrome (LQTS) and associated cardiac arrhythmias. As can be seen, none of the derivitives predicted to be hERG1 inhibitors.

## Conclusion

Regarding the important role of tyrosinase in the melanogenesis process, a series of 3,5-dihydroxybenzoyl-hydrazineylidene derivatives, **11a-n**, was designed and synthesized as tyrosinase inhibitor. The structure of all derivatives was comprehensively characterized using various spectroscopic data and elemental analysis. The in vitro tyrosinase inhibitory activity of these compounds revealed promising results, with most derivatives displaying moderate to good potency. Remarkable inhibitory potential was exhibited by compound **11m**, with an IC_50_ value of 55.39 ± 4.93 µM. A competitive mode of inhibition was indicated by kinetic analysis of the most potent analogs, with a *K*_*i*_ of 52.77 μM calculated for **11m.**

Furthermore, the molecular docking study exhibited significant interactions of **11m** with critical and highly conserved His amino acid and Cu^2+^ cofactors. Stability and consistent binding throughout a 100 ns simulation period were revealed by MD simulations of **11m** with tyrosinase. Notably, reduced fluctuations in critical residue regions compared to tyrosinase alone were indicated by the RMSD values of **11m**-tyrosinase complexes. Importantly, consistent interaction with Cu2 + cofactors was observed throughout the entire simulation period (100%). The enhanced potency of these compounds, particularly compound **11m**, was attributed to their chalcone-based structure with an amide linker and a terminal 4-methoxyphenyl tail moiety. The designed structure provides a promising foundation for developing potent tyrosinase inhibitors, which can be utilized as primary frameworks for forthcoming tyrosinase inhibitor development.

## Method and materials

### Chemistry

^1^H and ^13^C NMR spectra were recorded using a Bruker spectrometer 400 MHz instrument. Chemical shifts were reported in parts per million (ppm). Multiplicities were indicated by s (singlet), d (doublet), t (triplet), q (quartet), m (multiplet), and coupling constant *J* was reported in hertz (Hz). IR spectra were obtained with a Nicolet, FR -IR Magna 550. Melting points were also recorded using Kofler hot-stage apparatus. All the chemicals were purchased from Merck and Sigma.

#### Synthesis of methyl 3,5-dihydroxybenzoate (2)

3,5-Dihydroxybenzoic acid (10.00 g, 64.9 mmol) was dissolved in methanol (50 mL concentrated sulfuric acid (750 μL in 10 mL methanol) ) and the solution was allowed to reflux for 8 h. After cooling to ambient temperature, methanol was evaporated under reduced pressure, and the residue was dissolved in ethyl acetate (50 mL), which was then washed with saturated NaHCO_3_ solution and water. The organic layer was dried over anhydrous Na_2_SO_4,_ and ethyl acetate was removed in vacuo to afford methyl 3,5-dihydroxylbenzoate (10.74 g, 98%) as a white powder.

#### Synthesis of 3,5-dihydroxybenzohydrazide (3)

Methyl 3,5-dihydroxybenzoate (compound **2**, 1.060 mmol) was dissolved in absolute ethanol (5.0 mL) and hydrazine hydrate (0.5 mg, 15.625 mmol) was added. The reaction was allowed to react at room temperature for 16 h, concentrated, filtered, and dried. The intermediate 3,5-dihydroxybenzoic acid hydrazide was obtained.

#### Synthesis of Substituted 2-chloro-N-phenylacetamide (6a-n)

On an ice bath, chloroacetyl chloride (6.78 g, 0.06 mol) was added to a solution containing 0.050 mol of aniline derivatives dissolved in 40 mL of DMF. The resulting mixture was stirred at room temperature for 2 h, then poured into water and subsequently filtered to isolate compound **6a-n**^24,25^.

#### Synthesis of 4-(prop-2-yn-1-yloxy)benzaldehyde (9)

Propargyl bromide (23 mmol) was added to a solution containing 4-hydroxybenzaldehyde (compound 7, 23 mmol) and potassium carbonate (22 mmol) in 10 mL of dry DMF. The reaction mixture was stirred at room temperature for 2 h and then extracted with ethyl acetate to obtain crude O-propargyl benzaldehyde.

#### Synthesis of 2-(4-((4-formylphenoxy)methyl)-1H-1,2,3-triazol-1-yl)-N-phenylacetamide (10a-n)

At first, 2 mmol of different aryl acetamide derivatives (**6a-m**) and 2 mmol sodium azide in the presence of TEA were dissolved in DMF/H_2_O, and the mixture was refluxed for 3 h. The resulting solution was then refluxed for 3 h. Subsequently, 2 mmol of compound **9**, the catalytic quantity of CuSO_4_·5H_2_O, and sodium ascorbate were introduced. After 24 h, the products were synthesized, and the reaction's progress was monitored using TLC to ensure completion.

#### Synthesis of 11a-n derivatives

A mixture containing 0.020 mol of compounds **10a-n** and 0.021 mol of 3,5-dihydroxybenzoyl-hydrazineylidene, along with approximately 2 mL of acetic acid as the acid catalyst, was subjected to reflux in 96% ethanol (50 mL) for 8 h. Upon the completion of the reaction, the solvent was removed under reduced pressure, and the crude product was subsequently purified through crystallization in diethyl ether. After filtration and washing with diethyl ether, the product was left to dry at room temperature.

##### 2-(4-((4-((2-(3,5-dihydroxybenzoyl)hydrazineylidene)methyl)phenoxy)methyl)-1H-1,2,3-triazol-1-yl)-N-phenylacetamide (11a)

IR (KBr): 3521, 1679, 1364, 1196 cm^−1^. ^1^H NMR (400 MHz, ) δ 11.59 (s, 1H), 10.51 (s, 1H), 9.59 (s, 2H), 8.38 (s, 1H), 8.30 (s, 1H), 7.67 (dd, *J* = 8.8, 2.6 Hz, 2H), 7.33 (dt, *J* = 17.7, 7.3 Hz, 4H), 7.12 (dt, *J* = 23.1, 7.9 Hz, 3H), 6.74 (d, *J* = 2.1 Hz, 2H), 6.42 (d, *J* = 2.2 Hz, 1H), 5.28 (s, 2H), 5.21 (s, 2H).

^13^C NMR (101 MHz, DMSO) δ 164.64, 163.61, 160.02, 158.83, 147.75, 142.67, 138.87, 130.46, 129.40, 129.08, 128.84, 127.86, 126.91, 119.66, 115.52, 106.17, 105.97, 61.59, 52.69 ppm; *Anal*. Calcd for C_25_H_22_N_6_O_5_: C, 61.72; H, 4.56; N, 17.28; Found: C 61.66; H 4.35; N 17.49.

##### 2-(4-((4-((2-(3,5-dihydroxybenzoyl)hydrazono)methyl)phenoxy)methyl)-1H-1,2,3-triazol-1-yl)-N-(2-fluorophenyl)acetamide (11b)

IR (KBr): 3545, 1688, 1361, 1167, 768 cm^−1^. ^1^H NMR (400 MHz, DMSO-*d*_6_) δ 11.60 (s, 1H), 9.84 (s, 1H), 9.59 (s, 2H), 8.37 (s, 1H), 8.29 (s, 1H), 7.66 (d, *J* = 8.6 Hz, 2H), 7.15 (d, *J* = 8.6 Hz, 2H), 7.12 – 7.06 (m, 3H), 6.73 (d, *J* = 1.8 Hz, 2H), 6.41 (s, 1H), 5.41 (s, 2H), 5.24 (s, 2H). ^13^C NMR (101 MHz, DMSO-*d*_6_) δ 164.49, 163.65, 160.03, 158.95, 153.78, 149.95, 147.73, 142.65, 136.08, 135.55, 134.67, 129.08, 128.24, 127.76, 127.24, 126.86, 115.53, 106.15, 61.58, 52.09 ppm; *Anal*. Calcd for C_25_H_22_N_6_O_5_: C, 59.52; H, 4.20; N, 16.66; Found: C, 59.71; H, 4.34; N, 16.81.

##### 2-(4-((4-((2-(3,5-dihydroxybenzoyl)hydrazono)methyl)phenoxy)methyl)-1H-1,2,3-triazol-1-yl)-N-(4-fluorophenyl)acetamide (11c)

IR (KBr): 3541, 1681, 1358, 1171, 761 cm^−1^. ^1^H NMR (400 MHz, DMSO-*d*_6_) δ 11.59 (s, 1H), 10.70 (s, 1H), 9.62 (s, 2H), 8.38 (s, 1H), 8.30 (s, 1H), 7.67 (d, *J* = 8.4 Hz, 2H), 7.62 (d, *J* = 8.8 Hz, 2H), 7.41 (d, *J* = 8.8 Hz, 2H), 7.15 (d, *J* = 8.6 Hz, 2H), 6.74 (d, *J* = 1.6 Hz, 2H), 6.45 (s, 1H), 5.38 (s, 2H), 5.25 (s, 2H). ^13^C NMR (101 MHz, DMSO-*d*_6_) δ 164.87, 163.62, 161.22, 160.02, 158.84, 147.76, 142.72, 137.84, 136.11, 129.34, 129.09, 127.84, 126.92, 121.25, 115.53, 106.18, 61.58, 52.67 ppm; *Anal*. Calcd for C_25_H_21_FN_6_O_5_: C, 59.52; H, 4.20; N, 16.66; Found: C, 59.46; H, 4.02; N, 16.43.

##### N-(4-chlorophenyl)-2-(4-((4-((2-(3,5-dihydroxybenzoyl)hydrazineylidene)methyl)phenoxy)methyl)-1H-1,2,3-triazol-1-yl)Acetamide (11d)

IR (KBr): 3537, 1687, 1364, 1165, 758 cm^−1^. ^1^H NMR (400 MHz, DMSO-*d*_6_) δ 11.59 (s, 1H), 10.65 (s, 1H), 9.58 (s, 2H), 8.38 (s, 1H), 8.30 (s, 1H), 7.64 (dd, *J* = 18.2, 8.6 Hz, 4H), 7.46 – 7.34 (m, 2H), 7.15 (d, *J* = 8.4 Hz, 2H), 6.74 (d, *J* = 2.2 Hz, 2H), 6.42 (d, *J* = 2.2 Hz, 1H), 5.38 (s, 2H), 5.25 (s, 2H). ^13^C NMR (101 MHz, DMSO) δ 164.85, 163.61, 160.01, 158.83, 147.75, 142.71, 137.82, 136.09, 129.33, 129.08, 127.83, 127.75, 126.91, 121.24, 115.52, 106.17, 105.96, 61.58, 52.67. *Anal*. Calcd for C_25_H_21_ClN_6_O_5_: C, 57.64; H, 4.06; N, 16.13; Found: C 57.37; H 3.89; N 16.31.

##### N-(4-bromophenyl)-2-(4-((4-((2-(3,5-dihydroxybenzoyl)hydrazono)methyl)phenoxy)methyl)-1H-1,2,3-triazol-1-yl)acetamide (11e)

IR (KBr): 3537, 1658, 1556, 1253, 786 cm^−1^. ^1^H NMR (400 MHz, DMSO-*d*_6_) δ 11.59 (s, 1H), 10.61 (s, 1H), 9.58 (s, 2H), 8.38 (s, 1H), 8.30 (s, 1H), 7.67 (d, *J* = 8.4 Hz, 2H), 7.62 (d, *J* = 8.8 Hz, 2H), 7.41 (d, *J* = 8.8 Hz, 2H), 7.15 (d, *J* = 8.6 Hz, 2H), 6.76 – 6.72 (m, 2H), 6.42 (s, 1H), 5.38 (s, 2H), 5.25 (s, 2H) ppm; ^13^C NMR (101 MHz, DMSO-*d*_6_) δ 166.09, 163.62, 161.68, 158.84, 147.76, 142.72, 137.84, 136.11, 129.34, 129.09, 127.84, 126.92, 121.25, 115.53, 106.18, 61.58, 52.67 ppm; *Anal*. Calcd for C_25_H_21_BrN_6_O_5_: C, 53.11; H, 3.74; N, 14.86; Found: C, 52.97; H, 3.56; N, 14.62.

##### N-(2,4-dichlorophenyl)-2-(4-((4-((2-(3,5-dihydroxybenzoyl)hydrazineylidene)methyl)phenoxy)methyl)-1H-1,2,3-triazol-1-yl)acetamide (11f.)

IR (KBr): 3532, 1651, 1552, 1249, 792 cm^−1^. ^1^H NMR (400 MHz, DMSO-*d*_6_) δ 11.58 (s, 1H), 9.80 (s, 1H), 9.58 (s, 2H), 8.37 (s, 1H), 8.29 (s, 1H), 7.66 (d, *J* = 8.4 Hz, 2H), 7.15 (d, *J* = 8.4 Hz, 2H), 7.09 (d, *J* = 1.1 Hz, 3H), 6.73 (d, *J* = 2.1 Hz, 2H), 6.41 (d, *J* = 2.3 Hz, 1H), 5.41 (s, 2H), 5.24 (s, 2H), 2.16 (s, 6H). ^13^C NMR (101 MHz, DMSO) δ 164.47, 163.58, 160.03, 158.83, 147.71, 142.63, 136.09, 133.59, 131.20, 130.32, 129.07, 128.23, 127.74, 127.23, 126.84, 122.58, 115.51, 106.16, 105.70, 61.57, 52.09 ppm; *Anal*. Calcd for C_25_H_20_Cl_2_N_6_O_6_: C, 54.07; H, 3.63; N, 15.13; Found: C 53.89; H 3.47; N 15.02.

##### N-(2,6-dichlorophenyl)-2-(4-((4-((2-(3,5-dihydroxybenzoyl)hydrazineylidene)methyl)phenoxy)methyl)-1H-1,2,3-triazol-1-yl)Acetamide (11g)

IR (KBr): 3533, 1655, 1325, 1270, 802 cm^−1^. ^1^H NMR (400 MHz, DMSO-*d*_6_) δ 11.57 (s, 1H), 9.80 (s, 1H), 9.61 (s, 1H), 8.37 (s, 1H), 8.29 (s, 1H), 7.66 (d, *J* = 8.3 Hz, 2H), 7.12 (d, *J* = 28.2 Hz, 5H), 6.73 (d, *J* = 2.3 Hz, 2H), 6.41 (d, *J* = 2.2 Hz, 1H), 5.41 (s, 2H), 5.24 (s, 2H). ^13^C NMR (101 MHz, DMSO) δ 164.47, 163.63, 160.01, 158.93, 147.72, 142.64, 136.06, 135.54, 134.65, 129.07, 128.23, 127.75, 127.23, 126.85, 115.51, 106.12, 105.99, 61.57, 52.09 ppm; *Anal*. Calcd for C_25_H_20_Cl_2_N_6_O_5_: C, 54.07; H, 3.63; N, 15.13; Found: C 54.16; H 3.36; N 15.35.

##### 2-(4-((4-((2-(3,5-dihydroxybenzoyl)hydrazineylidene)methyl)phenoxy)methyl)-1H-1,2,3-triazol-1-yl)-N-(4-nitrophenyl)Acetamide (11h)

IR (KBr): 3539, 1696, 1551, 1347, 1228 cm^−1^. ^1^H NMR (400 MHz, ) δ 11.58 (s, 1H), 11.02 (s, 1H), 9.58 (s, 2H), 8.61 (d, *J* = 2.2 Hz, 2H), 8.33 (d, *J* = 8.4 Hz, 2H), 7.97 (dd, *J* = 8.2, 2.3 Hz, 2H), 7.90 (d, *J* = 8.6 Hz, 3H), 7.67 (d, *J* = 8.3 Hz, 3H), 7.28 (d, *J* = 8.5 Hz, 2H), 6.74 (d, *J* = 2.2 Hz, 1H), 6.42 (d, *J* = 2.2 Hz, 1H), 5.34 (s, 2H), 5.26 (s, 2H); ^13^C NMR (101 MHz, DMSO) δ 164.43, 163.47, 160.55, 158.83, 148.46, 147.53, 144.81, 142.36, 139.93, 129.09, 127.48, 126.43, 125.69, 118.84, 115.67, 106.03, 105.41, 61.88, 52.69 ppm; *Anal*. Calcd for C_25_H_21_N_7_O_7_: C, 56.50; H, 3.98; N, 18.45; Found: C 56.27; H 4.21; N 18.62.

##### 2-(4-((4-((2-(3,5-dihydroxybenzoyl)hydrazineylidene)methyl)phenoxy)methyl)-1H-1,2,3-triazol-1-yl)-N-(2-methyl-4-nitrophenyl)acetamide (11i)

IR (KBr): 3527, 1690, 1551, 1352, 1205 cm^−1^. ^1^H NMR (400 MHz, ) δ 11.58 (s, 1H), 9.89 (s, 1H), 9.58 (s, 2H), 8.38 (s, 1H), 8.32 (s, 1H), 8.18 (d, *J* = 2.6 Hz, 1H), 7.96 (dd, *J* = 8.9, 3.1 Hz, 1H), 7.90 (d, *J* = 8.7 Hz, 1H), 7.67 (d, *J* = 8.5 Hz, 2H), 7.15 (d, *J* = 8.5 Hz, 2H), 6.73 (d, *J* = 2.1 Hz, 2H), 6.42 (d, *J* = 2.3 Hz, 1H), 5.33 (s, 2H), 5.26 (s, 2H), 2.42 (s, 3H). ^13^C NMR (101 MHz, DMSO) δ 164.39, 163.85, 160.88, 158.17, 147.06, 143.95, 142.21, 139.83, 136.35, 132.28, 129.08, 127.35, 126.04, 123.72, 120.44, 115.52, 109.84, 106.27, 105.87, 61.12, 52.63, 18.32. *Anal*. Calcd for C_26_H_23_N_7_O_7_: C, 57.25; H, 4.25; N, 17.97; Found: C 57.10; H 4.14; N 18.09.

##### 2-(4-((4-((2-(3,5-dihydroxybenzoyl)hydrazono)methyl)phenoxy)methyl)-1H-1,2,3-triazol-1-yl)-N-(p-tolyl)acetamide (11j)

IR (KBr): 3448, 1671, 1389, 1231 cm^−1^. ^1^H NMR (400 MHz, DMSO-*d*_6_) δ 11.59 (s, 1H), 10.44 (s, 1H), 9.61 (s, 1H), 8.38 (s, 1H), 8.29 (s, 1H), 7.67 (d, *J* = 8.5 Hz, 2H), 7.50 (d, *J* = 8.3 Hz, 2H), 7.16 (t, *J* = 7.9 Hz, 4H), 6.87 – 6.61 (m, 2H), 6.42 (s, 1H), 5.35 (s, 2H), 5.25 (s, 2H) ), 2.31 (s, 3H). ^13^C NMR (101 MHz, DMSO-*d*_6_) δ 164.10, 161.35, 160.03, 158.84, 156.00, 147.75, 142.66, 136.10, 131.97, 129.09, 127.75, 126.90, 121.22, 115.53, 114.49, 106.17, 61.59, 52.61, 20.80 ppm; *Anal*. Calcd for C_26_H_24_N_6_O_5_: C, 62.39; H, 4.83; N, 16.79; Found: C, 62.25; H, 4.63; N, 16.64.

##### 2-(4-((4-((2-(3,5-dihydroxybenzoyl)hydrazineylidene)methyl)phenoxy)methyl)-1H-1,2,3-triazol-1-yl)-N-(2,6-dimethylphenyl)Acetamide (11k)

IR (KBr): 3562, 1678, 1341, 1210 cm^−1^. ^1^H NMR (400 MHz, ) δ 11.58 (s, 1H), 10.25 (s, 0H), 9.58 (s, 2H), 8.37 (s, 1H), 8.29 (s, 1H), 7.66 (d, *J* = 8.4 Hz, 2H), 7.09 (s, 8H), 6.73 (d, *J* = 2.2 Hz, 2H), 6.42 (t, *J* = 2.2 Hz, 1H), 5.41 (s, 3H), 5.24 (s, 2H), 2.16 (s, 6H) ppm; ^13^C NMR (101 MHz, DMSO) δ 164.47, 163.59, 160.01, 158.82, 147.73, 142.63, 137.87, 136.09, 130.50, 129.07, 128.23, 127.23, 126.85, 126.76, 115.51, 106.16, 105.59, 61.46, 52.08, 18.50 ppm; *Anal*. Calcd for C_27_H_26_N_6_O_5_: C, 63.03; H, 5.09; N, 16.33; Found: C 63.28; H 5.14; N 16.19.

##### 2-(4-((4-((2-(3,5-dihydroxybenzoyl)hydrazineylidene)methyl)phenoxy)methyl)-1H-1,2,3-triazol-1-yl)-N-(4-ethylphenyl)acetamide (11l)

IR (KBr): 3544, 1677, 1330, 1171 cm^−1^. ^1^H NMR (400 MHz, DMSO-*d*_6_) δ 11.59 (s, 1H), 10.44 (s, 1H), 9.61 (s, 2H), 8.38 (s, 1H), 8.29 (s, 1H), 7.67 (d, *J* = 8.4 Hz, 2H), 7.56 – 7.45 (m, 2H), 7.16 (t, *J* = 7.9 Hz, 4H), 6.74 (d, *J* = 2.1 Hz, 2H), 6.42 (d, *J* = 2.3 Hz, 1H), 5.35 (s, 2H), 5.25 (s, 2H), 2.56 (q, *J* = 7.6 Hz, 2H), 1.16 (t, *J* = 7.6 Hz, 3H) ppm; ^13^C NMR (101 MHz, DMSO) δ 164.37, 163.61, 160.02, 158.85, 147.75, 142.65, 139.66, 136.54, 129.08, 128.57, 127.75, 126.90, 122.87, 119.75, 115.52, 106.17, 105.97, 61.58, 52.66, 28.05, 16.11 ppm; *Anal*. Calcd for C_27_H_26_N_6_O_5_: C, 63.03; H, 5.09; N, 16.33; Found: C 62.84; H 5.26; N 16.52.

##### 2-(4-((4-((2-(3,5-dihydroxybenzoyl)hydrazineylidene)methyl)phenoxy)methyl)-1H-1,2,3-triazol-1-yl)-N-(4-methoxyphenyl)acetamide (11m)

IR (KBr): 3442, 1678, 1383, 1235 cm^−1^. ^1^H NMR (400 MHz, DMSO-*d*_6_) δ 11.59 (s, 1H), 10.37 (s, 1H), 9.59 (s, 2H), 8.38 (s, 1H), 8.29 (s, 1H), 7.67 (d, *J* = 8.4 Hz, 2H), 7.54 – 7.46 (m, 2H), 7.15 (d, *J* = 8.5 Hz, 2H), 6.97 – 6.86 (m, 2H), 6.74 (d, *J* = 2.1 Hz, 2H), 6.41 (d, *J* = 2.2 Hz, 1H), 5.33 (s, 2H), 5.25 (s, 2H), 3.73 (s, 3H). ^13^C NMR (101 MHz, DMSO-*d*_6_) δ 164.09, 163.60, 160.02, 158.83, 155.98, 147.74, 142.64, 136.09, 131.96, 129.08, 127.74, 126.89, 121.21, 115.52, 114.48, 106.16, 105.95, 61.58, 55.62, 52.60 ppm; *Anal*. Calcd for C_26_H_24_N_6_O_6_: C, 60.46; H, 4.68; N, 16.27; Found: C 60.63; H 4.47; N 16.01.

##### N-benzyl-2-(4-((4-((2-(3,5-dihydroxybenzoyl)hydrazineylidene)methyl)phenoxy)methyl)-1H-1,2,3-triazol-1-yl)acetamide (11n)

IR (KBr): 3445, 1668, 1328, 1212 cm^−1^. ^1^H NMR (400 MHz, DMSO-*d*_6_) δ 11.58 (s, 1H), 9.58 (s, 2H), 8.87 (t, *J* = 5.9 Hz, 1H), 8.38 (s, 1H), 8.24 (s, 1H), 7.66 (d, *J* = 8.4 Hz, 2H), 7.38 – 7.23 (m, 5H), 7.15 (t, *J* = 6.8 Hz, 3H), 6.73 (d, *J* = 2.1 Hz, 2H), 6.42 (t, *J* = 2.2 Hz, 1H), 5.23 (s, 2H), 5.21 (s, 2H), 4.34 (d, *J* = 5.8 Hz, 2H). ^13^C NMR (101 MHz, DMSO) δ 165.87, 163.59, 160.02, 158.82, 147.73, 142.58, 139.16, 136.09, 129.37, 129.07, 128.84, 127.86, 127.48, 126.79, 115.51, 106.16, 105.94, 61.58, 52.07, 42.84. *Anal*. Calcd for C_26_H_24_N_6_O_5_: C, 62.39; H, 4.83; N, 16.79; Found: C 62.11; H 4.62; N 16.57.

### Tyrosinase inhibitory assay

The tyrosinase inhibitory activities of derivatives were performed according to the previously reported procedures^26,27^. All the test samples were first dissolved in DMSO at dilution to the required final concentrations. Initially, in a 96-well microplate, 10 µl of test samples were added to 160 µl of phosphate buffer (pH = 6.8), and then 10 µl tyrosinase was added. After the mixture was pre-incubated at 28 °C for 20 min, 20 µl of L-DOPA solution was added. DMSO without test compounds was used as the control, and kojic acid was used as a positive control. After 8 min incubation absorbance of samples was measured at 490 nm. Each assay was conducted as three separate replicates.

### Enzyme kinetic studies

The kinetic study for tyrosinase inhibition by **11m** as the most potent analog was carried out using four different concentrations of inhibitor (10, 20, 30, 50 and 70 µM) against tyrosinase with different concentrations of L-DOPA (0.25, 0.5, 0.75, and 1 mM) as the substrate. The Lineweaver–Burk reciprocal plot was provided by plotting 1/V against 1/[S] at variable concentrations of the L-DOPA.

### Molecular docking

The molecular docking studies were performed using the Maestro molecular modeling platform (version 10.5) by Schrödinger, LLC (Maestro, Schrödinger, LLC, New York, NY, 2021). The 3D crystal structure of tyrosinase was retrieved from the Protein Data Bank (PDB code: 2Y9X). Protein was prepared in which the water molecules and the cognate ligand (tropolone) were removed from the receptor and the hydrogen atoms were added and non-polar hydrogens were merged into related atoms of the receptor via protein preparation. To prepare the ligand, the 2D structures of the ligands were drawn in ChemDraw version 12, converted into SDF files, and subjected to the LigPrep module. Ligands were prepared by OPLS_2005 force field using EPIK. The derivative was docked on binding sites using induced-fit docking with a box size of 20 Å, reporting 10 poses per ligand to form the final complex. The other parameters were as follows: confirmation sampling with an energy window of 2.5 kcal/mol, receptor and ligand van der Waals scaling 0.5, and Glide redocking into receptor structures within 30 kcal/mol of the best structure, and all parameters were set as default^28,29^.

### MD simulation

The molecular simulation was conducted utilizing the Desmond of Schrödinger package (version 10.5, Maestro, Schrödinger, LLC, New York, NY, 2021). To prepare the system for MD simulation, the protein–ligand complexes were immersed in an orthorhombic box of suitable dimensions with periodic boundary conditions and solvated using explicit water molecules of the SPC type. Additionally, the system was neutralized by incorporating an appropriate number of counter-ions, and a 0.15 M solution of NaCl was employed to mimic realistic cellular ionic concentrations^20^. The MD protocol involved minimization, pre-production, and production MD simulation steps. Finally, the system was subjected to produce MD simulations for 100 ns for ao enzyme and protein–ligand complex. The systems' dynamic behavior and structural changes were analyzed by calculating the RMSD and RMSF^30^.

### Supplementary Information

Supplementary Information.

## Data Availability

The datasets generated and/or analysed during the current study are available in the Worldwide Protein Data Bank (wwPDB) repository. (https://www.rcsb.org/structure/2y9x).
