# Supplementary material for: Structure-based development of 3,5-dihydroxybenzoyl-hydrazineylidene as tyrosinase inhibitor; in vitro and in silico study
Source: Sci Rep. 2024 Jan 17;14:1540. doi: 10.1038/s41598-024-52022-6 (PMC10794188; doi:10.1038/s41598-024-52022-6)
Supplement: Supplementary file 1 — Supplementary Information. [file 41598_2024_52022_MOESM1_ESM.docx]

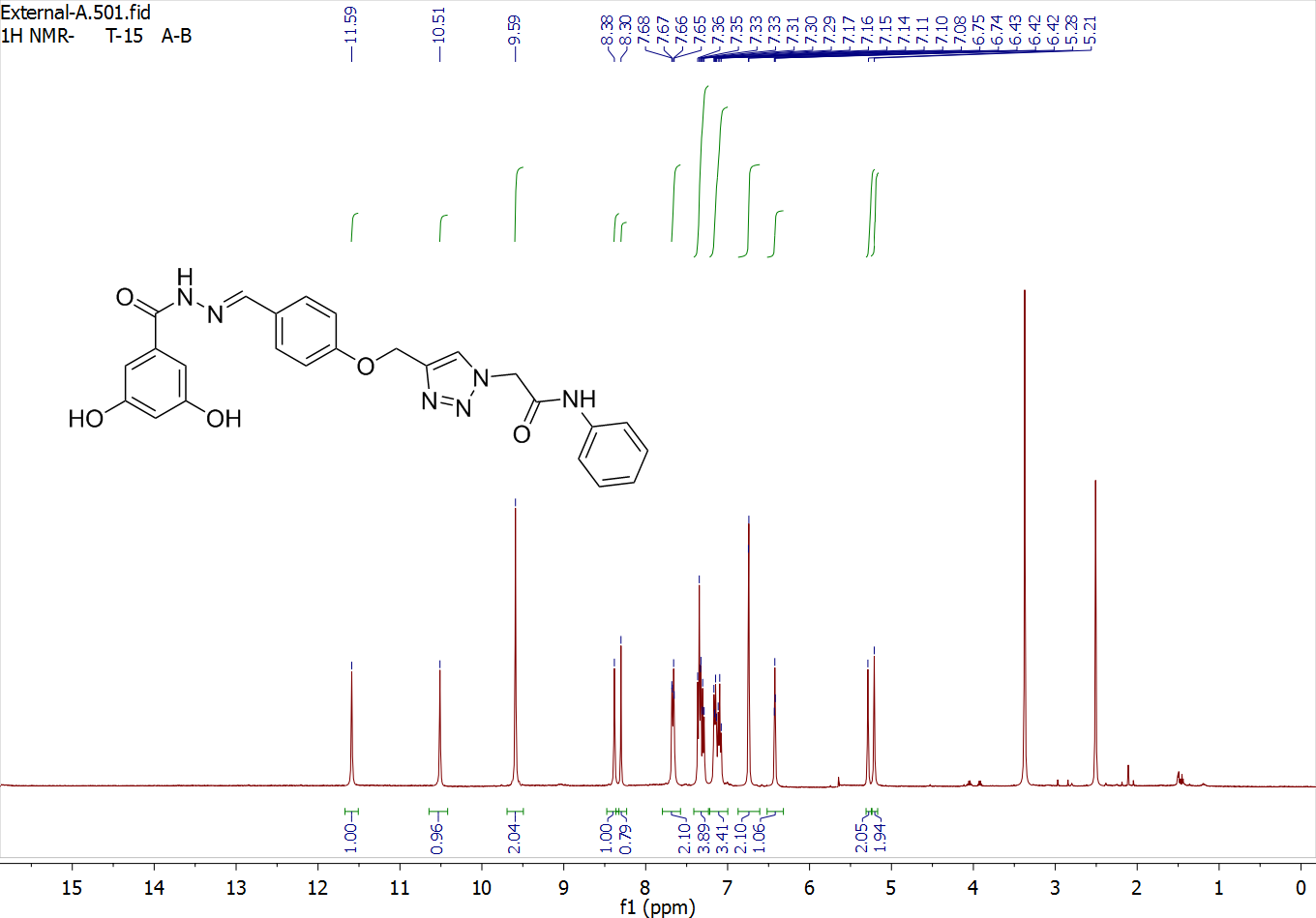


**Fig. S1.** ^1^H-NMR of **11a**


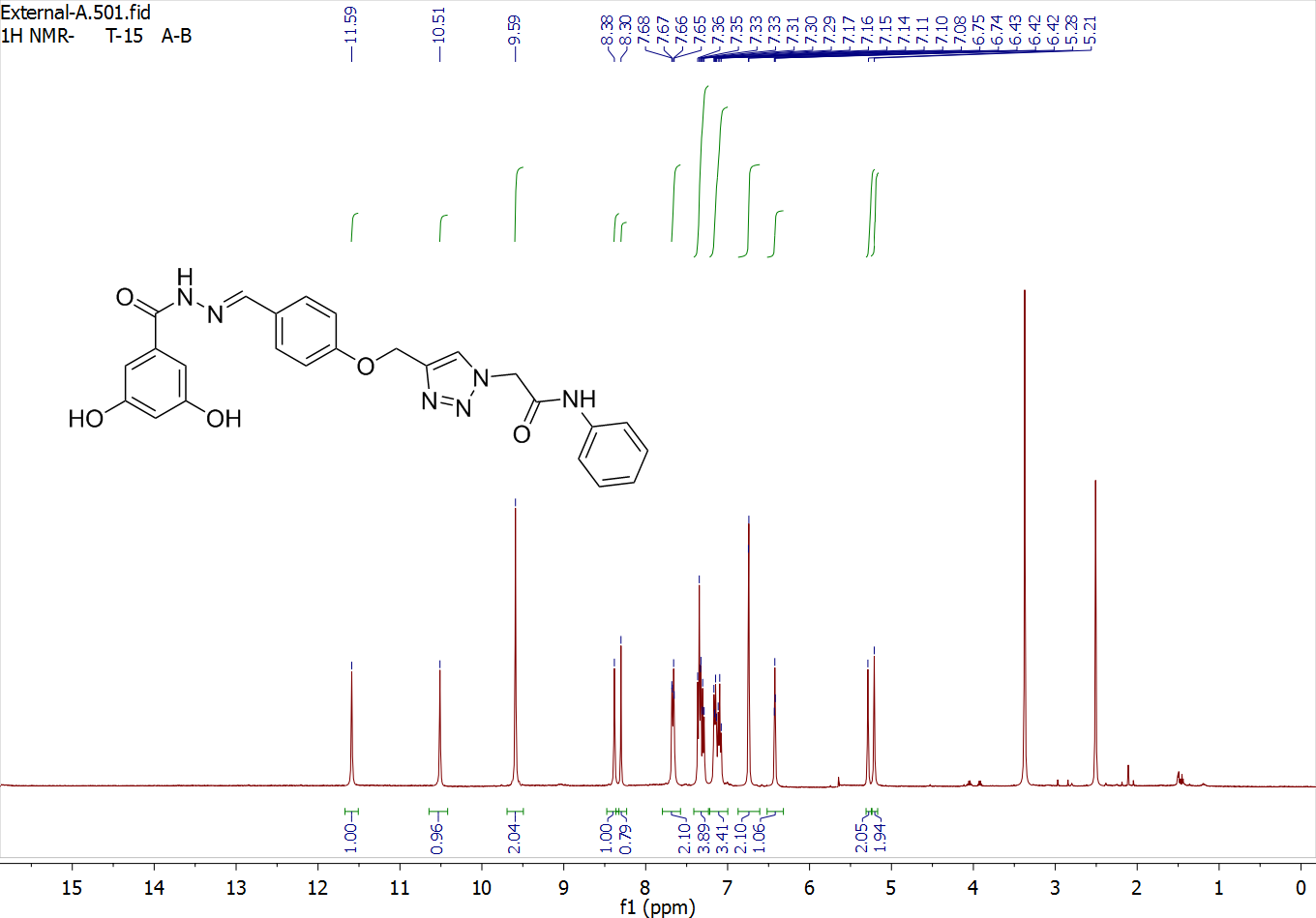


**Fig. S2.** ^13^C-NMR of **11a**


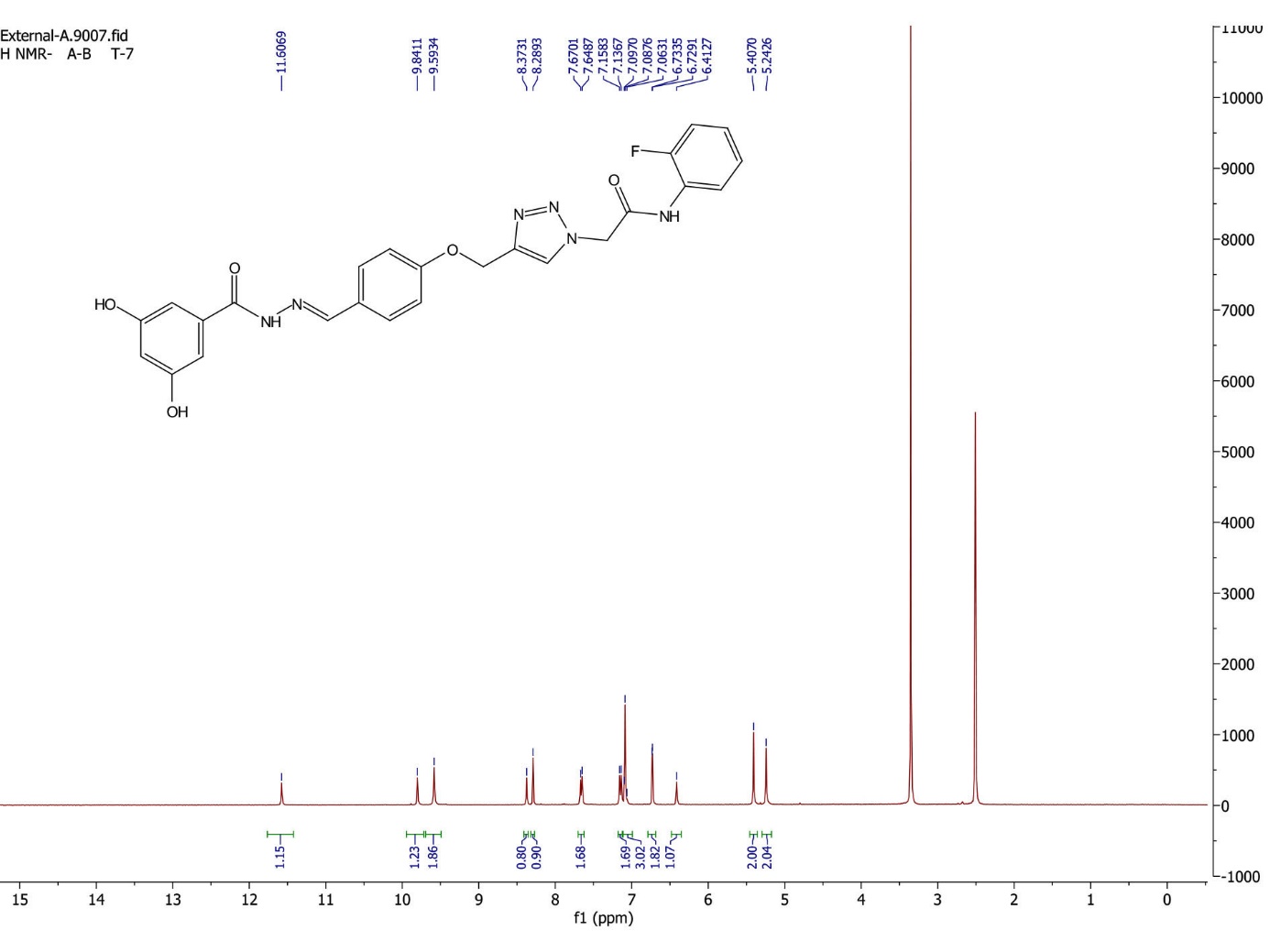


**Fig. S3.** ^1^H-NMR of **11b**


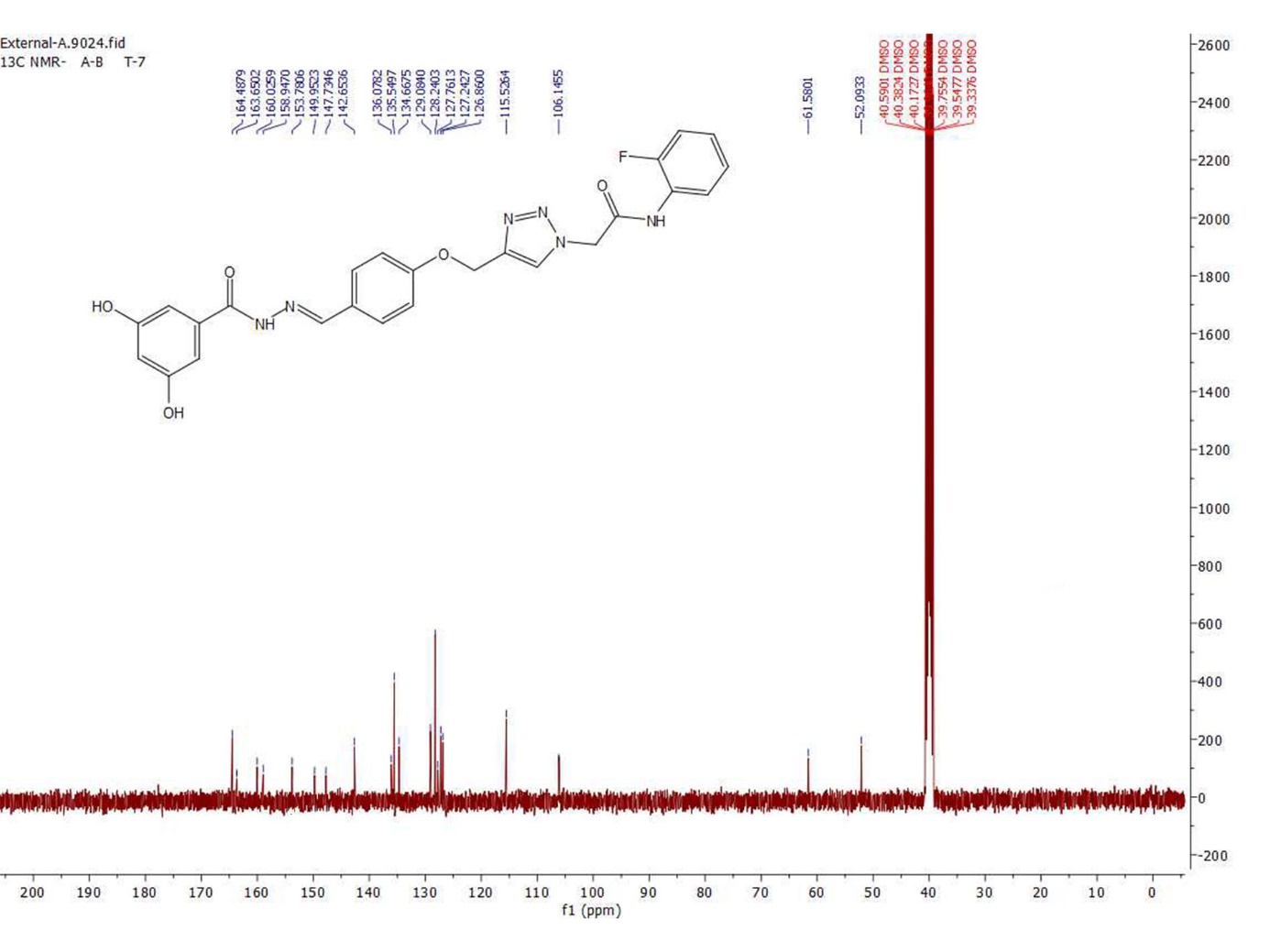


**Fig. S4.** ^13^C-NMR of **11b**


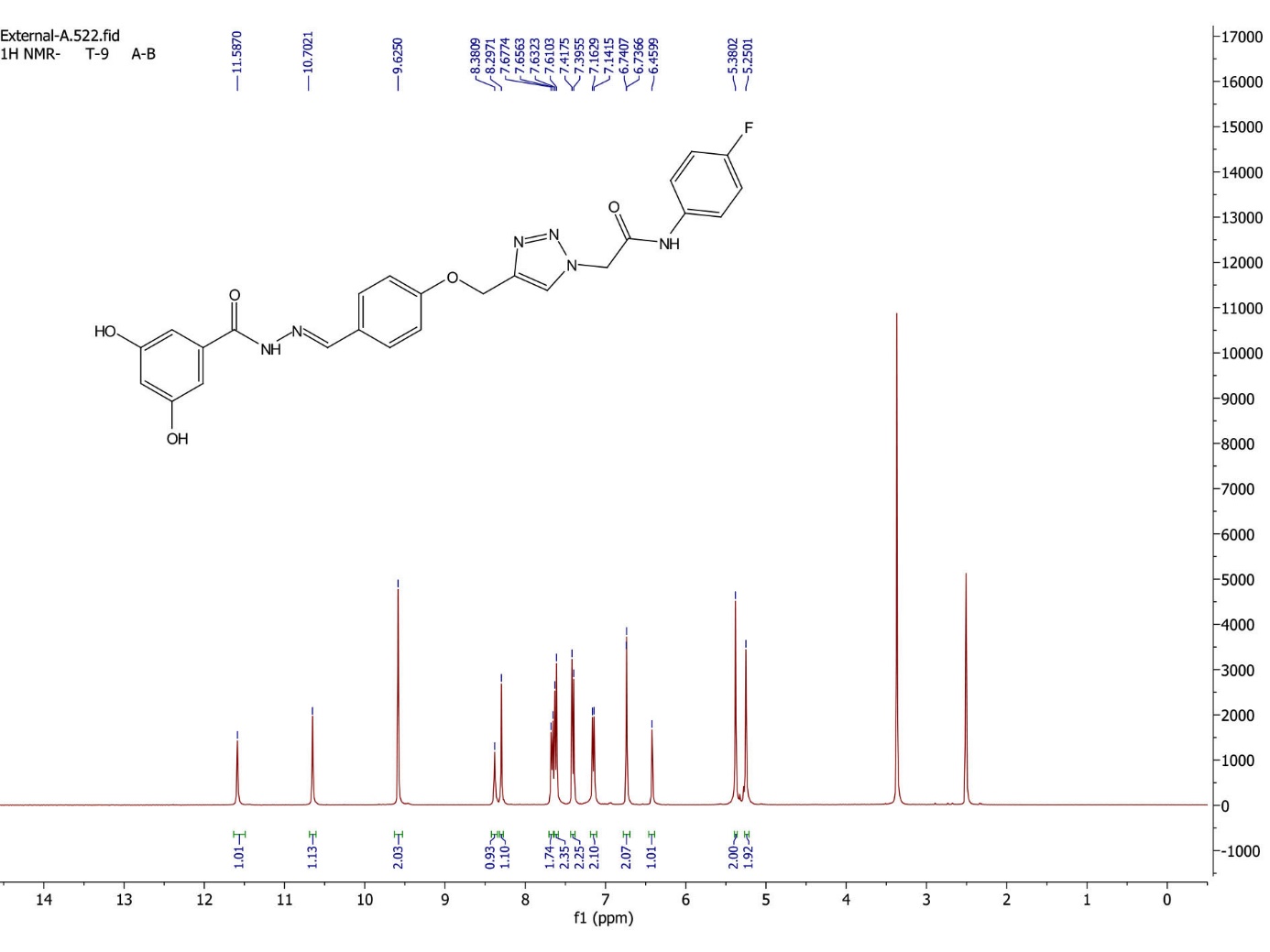


**Fig. S5.** ^1^H-NMR of **11c**


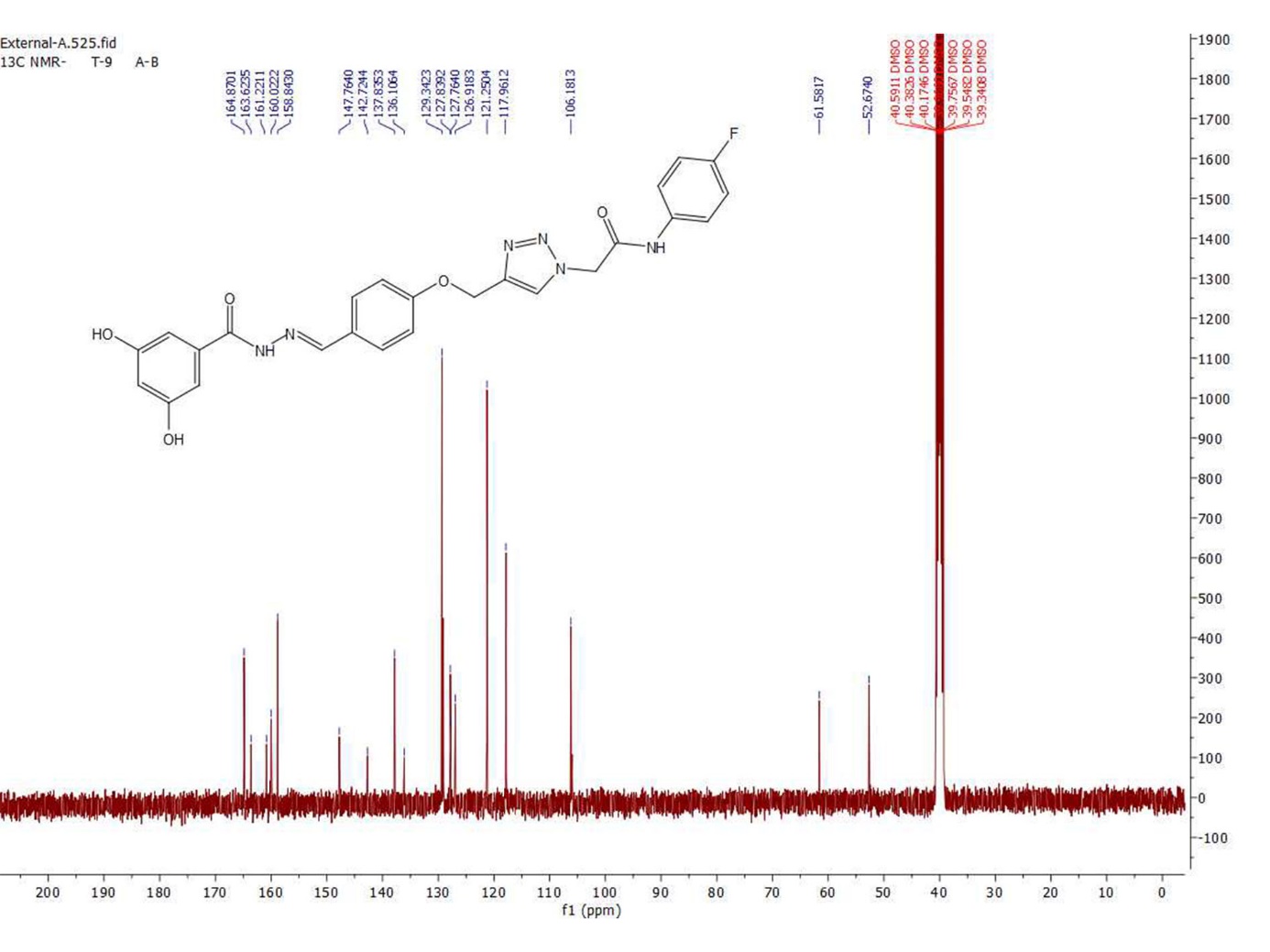


**Fig. S6.** ^13^C-NMR of **11c**


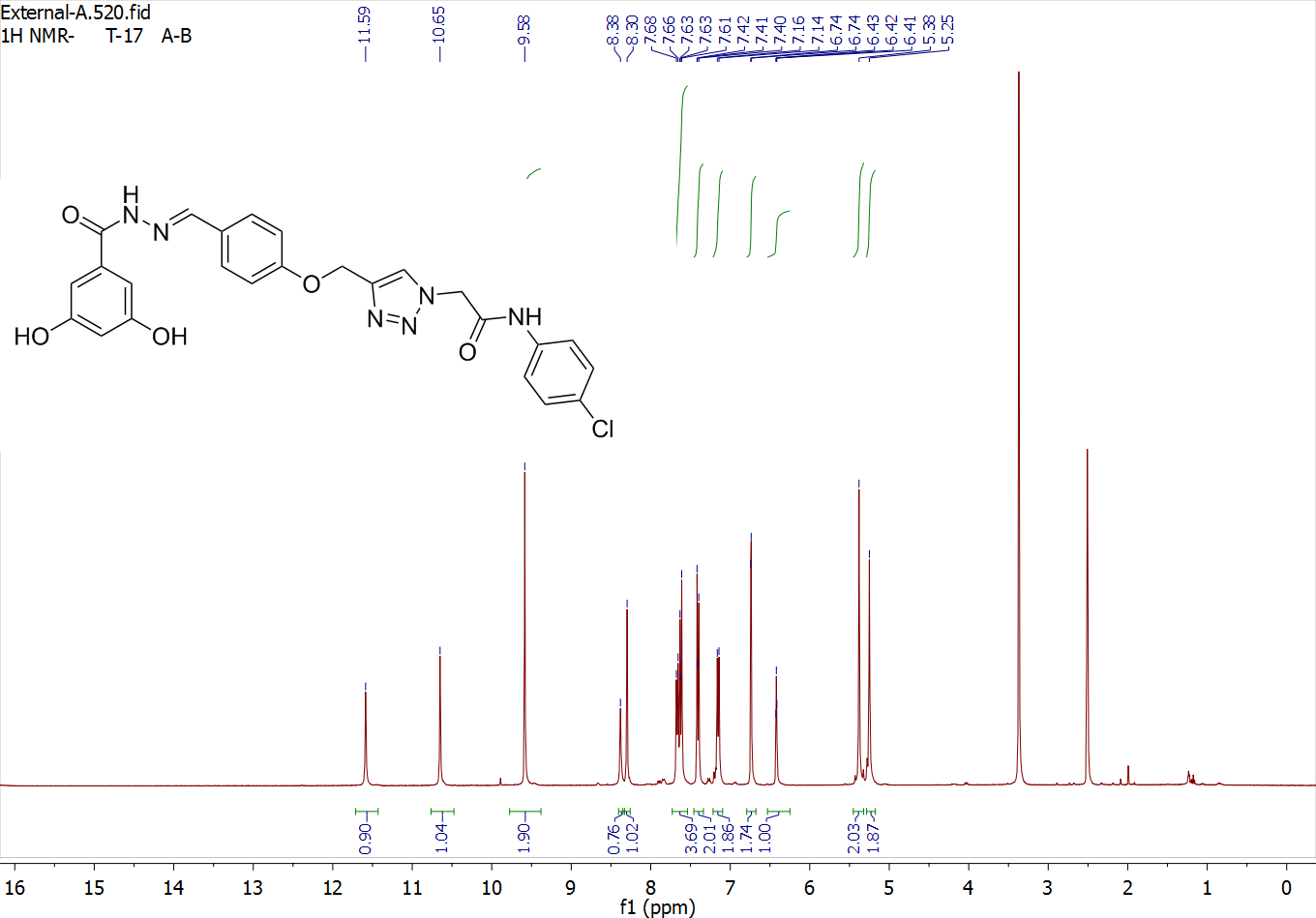


**Fig. S7.** ^1^H-NMR of **11d**


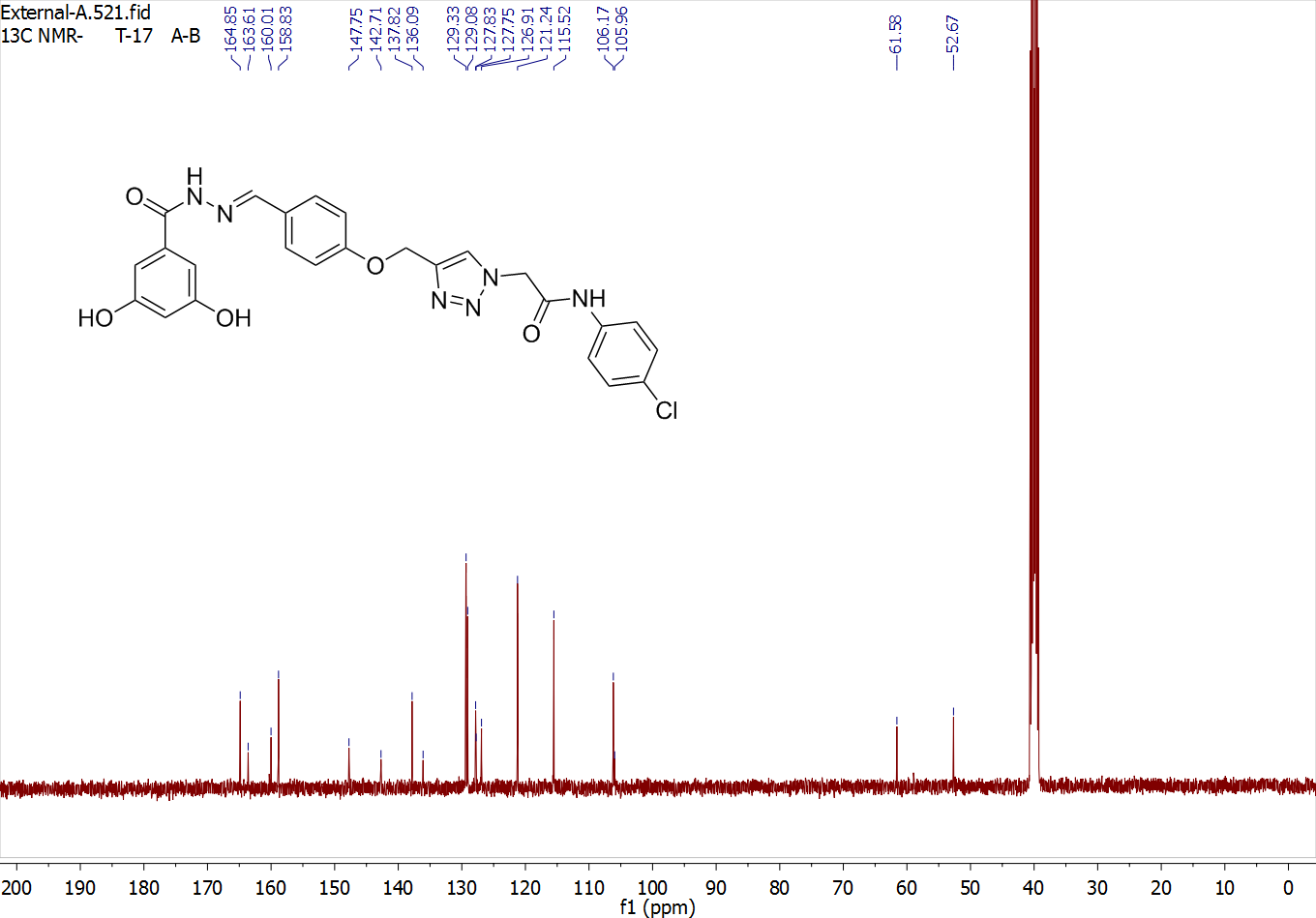


**Fig. S8.** ^1^H-NMR of **11d**


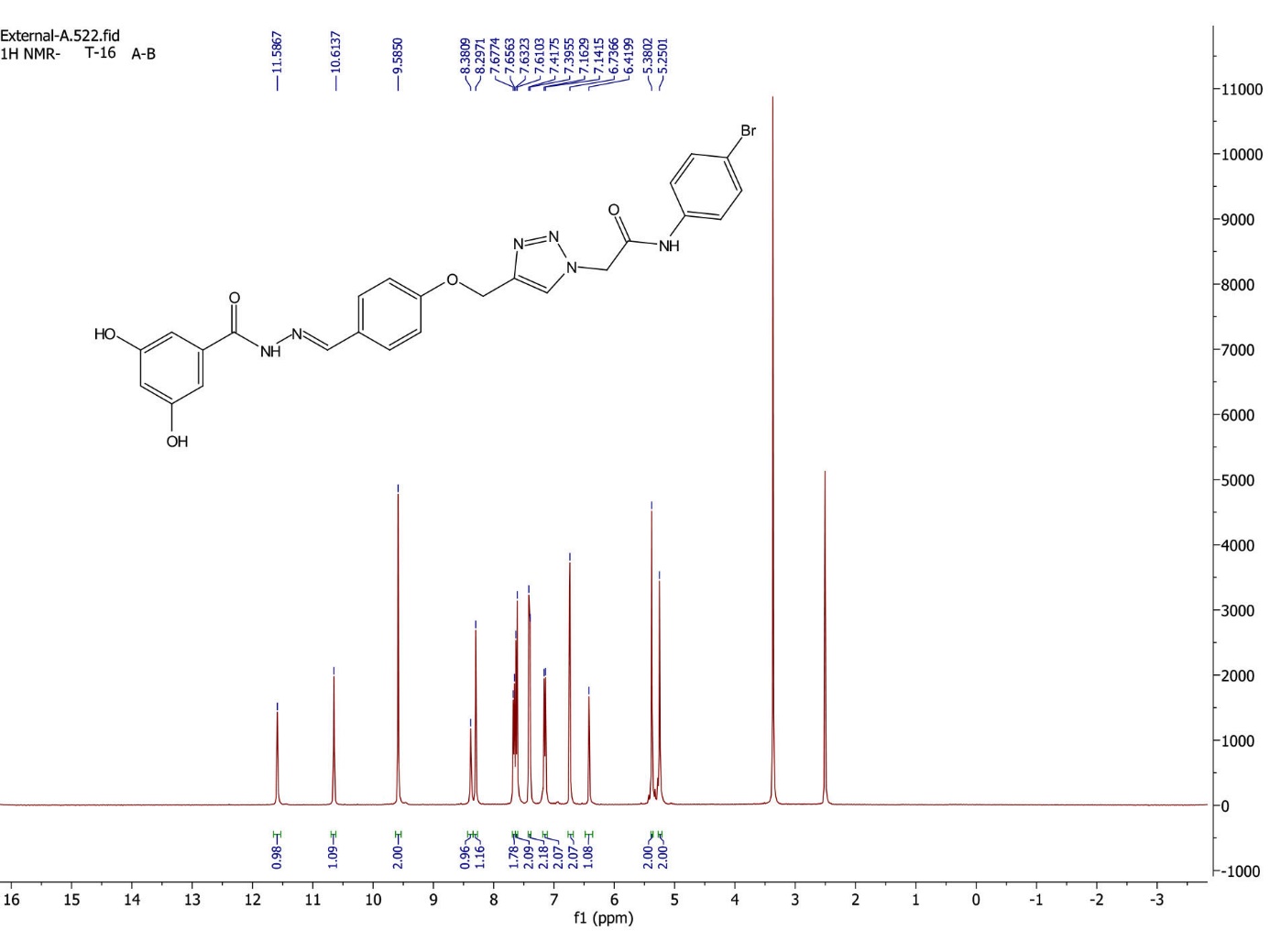


**Fig. S9.** ^1^H-NMR of **11e**


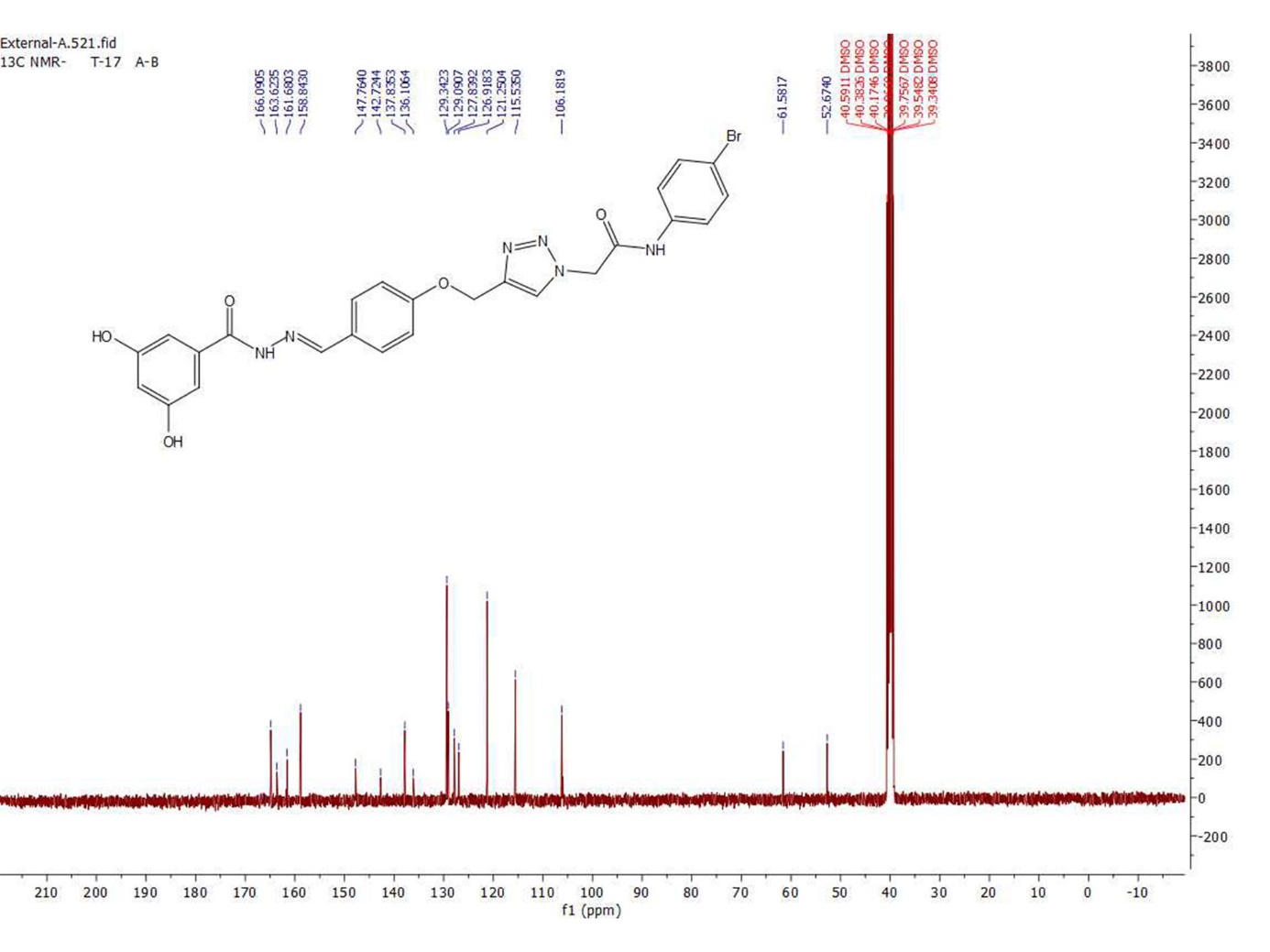


**Fig. S10.** ^13^C-NMR of **11e**


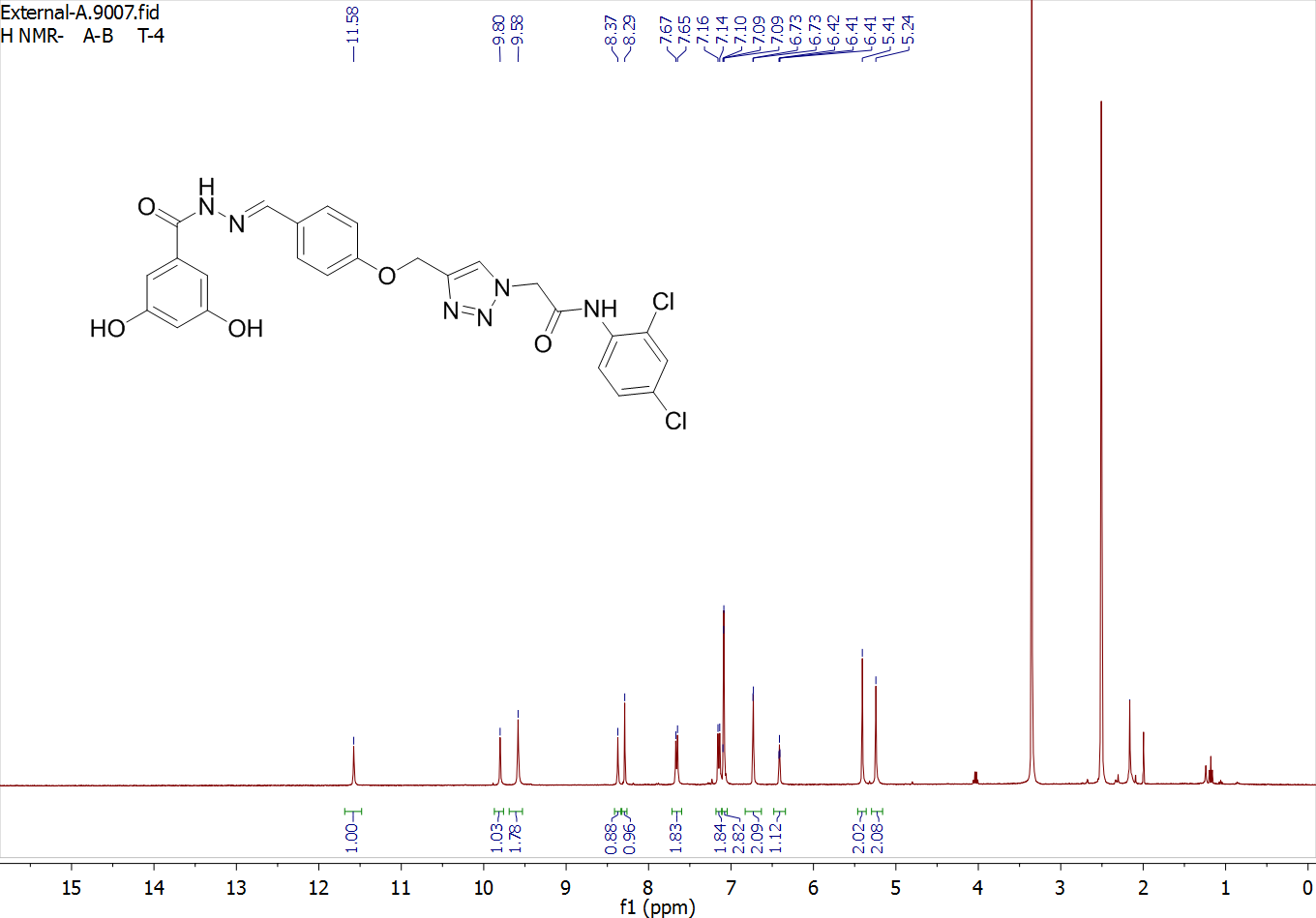


**Fig. S11.** ^1^H-NMR of **11f**


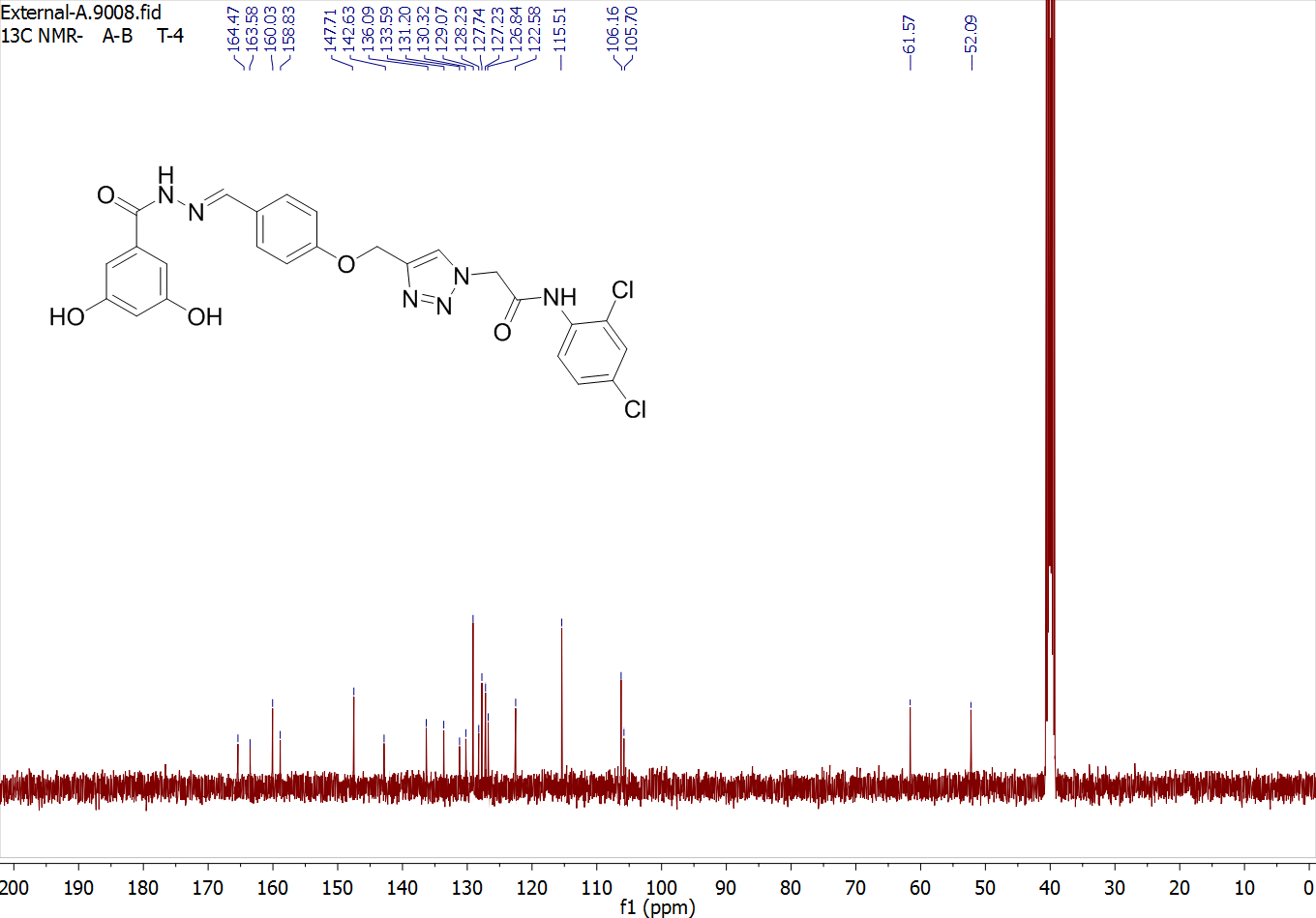


**Fig. S12.** ^13^C-NMR of **11f**


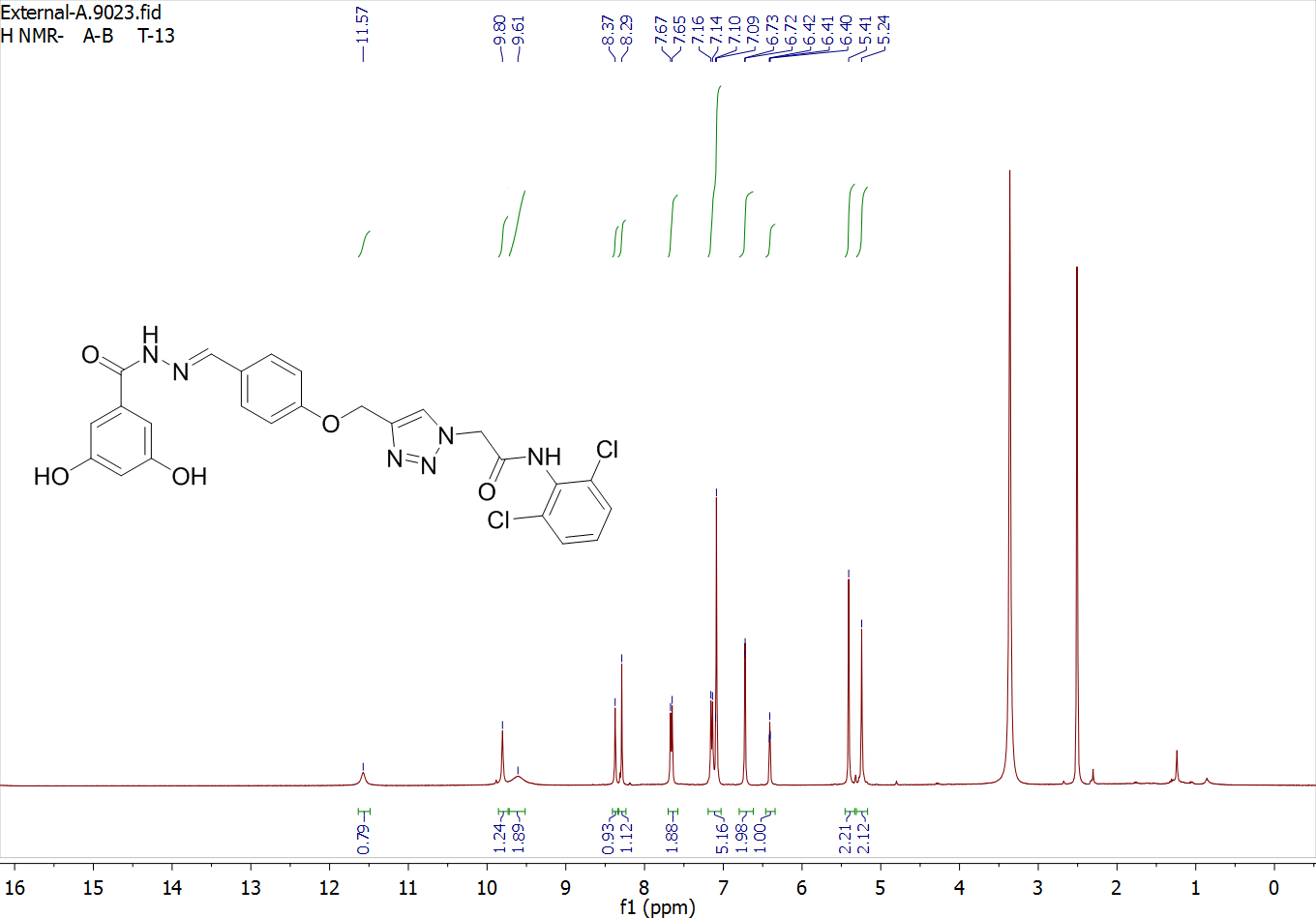


**Fig. S13.** ^1^H-NMR of **11g**


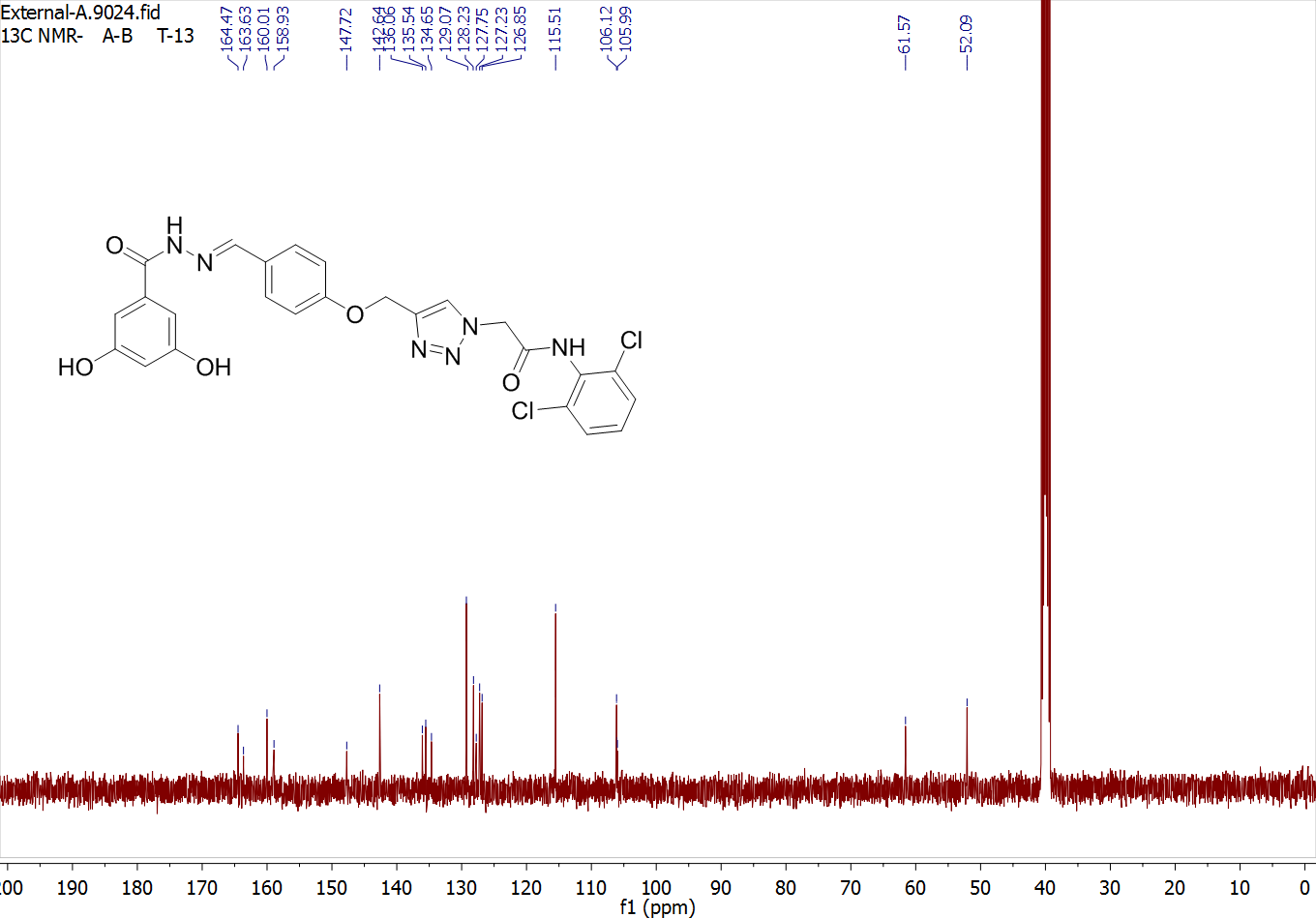


**Fig. S14.** ^13^C-NMR of **11g**


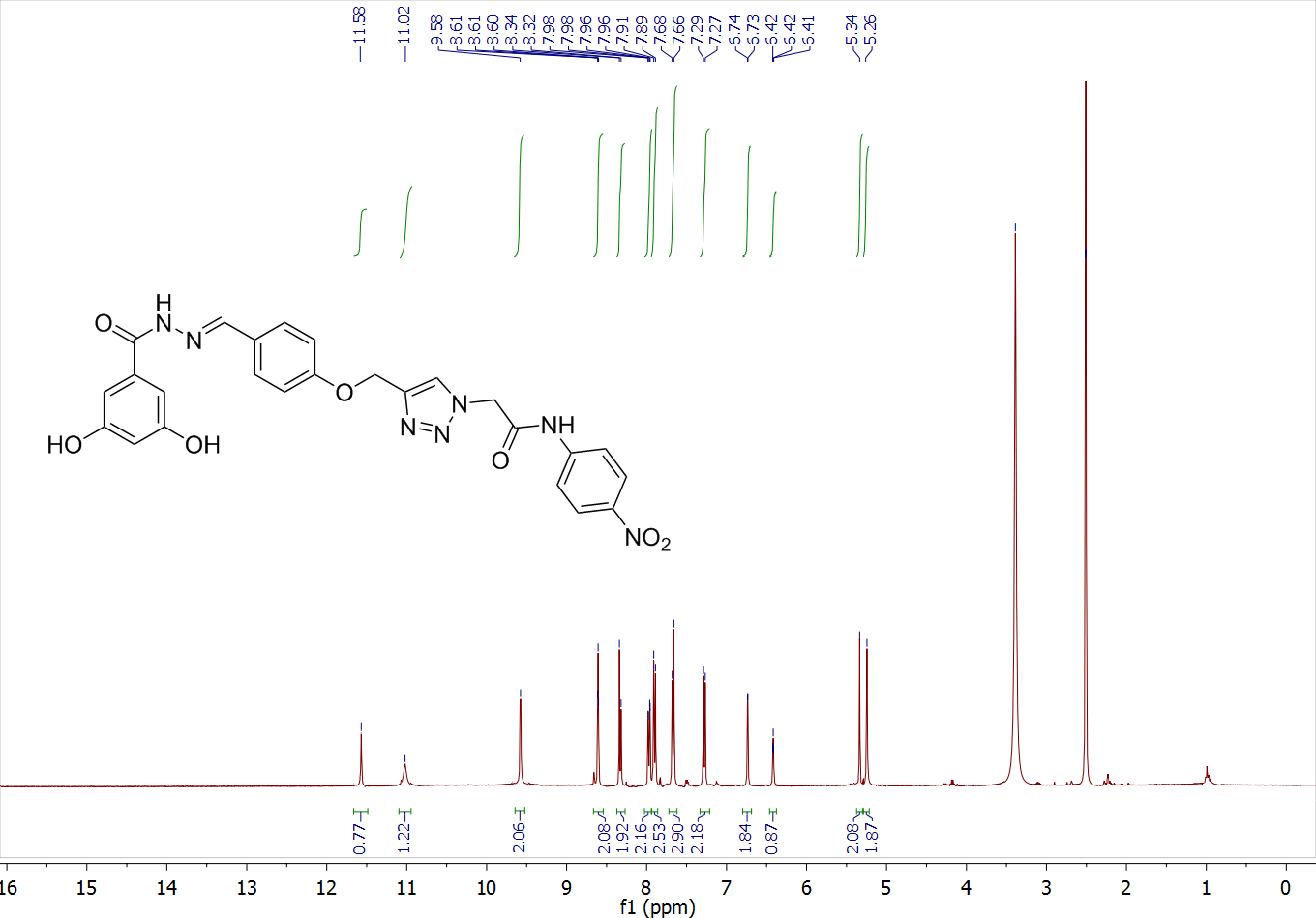


**Fig. S15.** ^1^H-NMR of **11h**


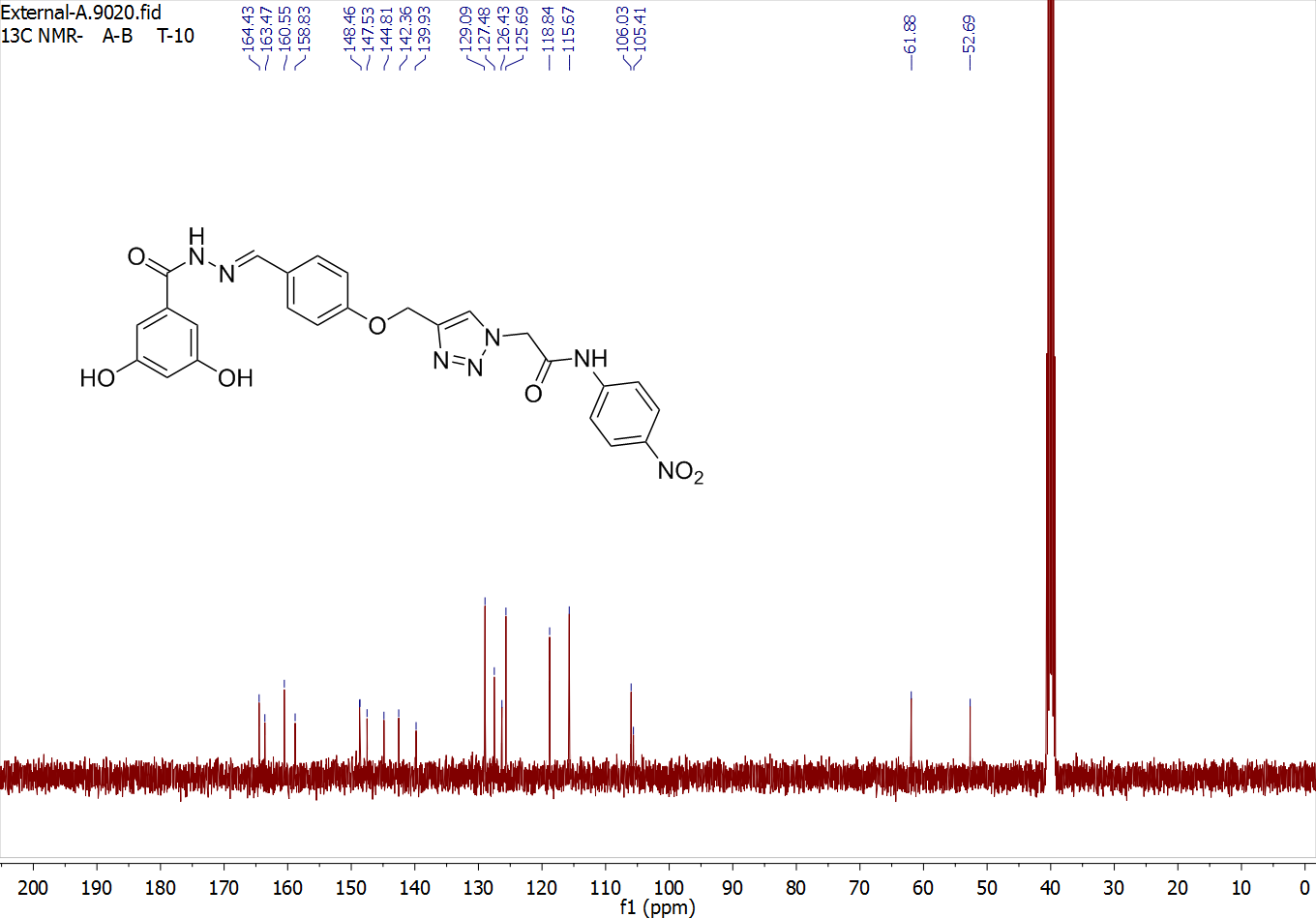


**Fig. S16.** ^13^C-NMR of **11h**


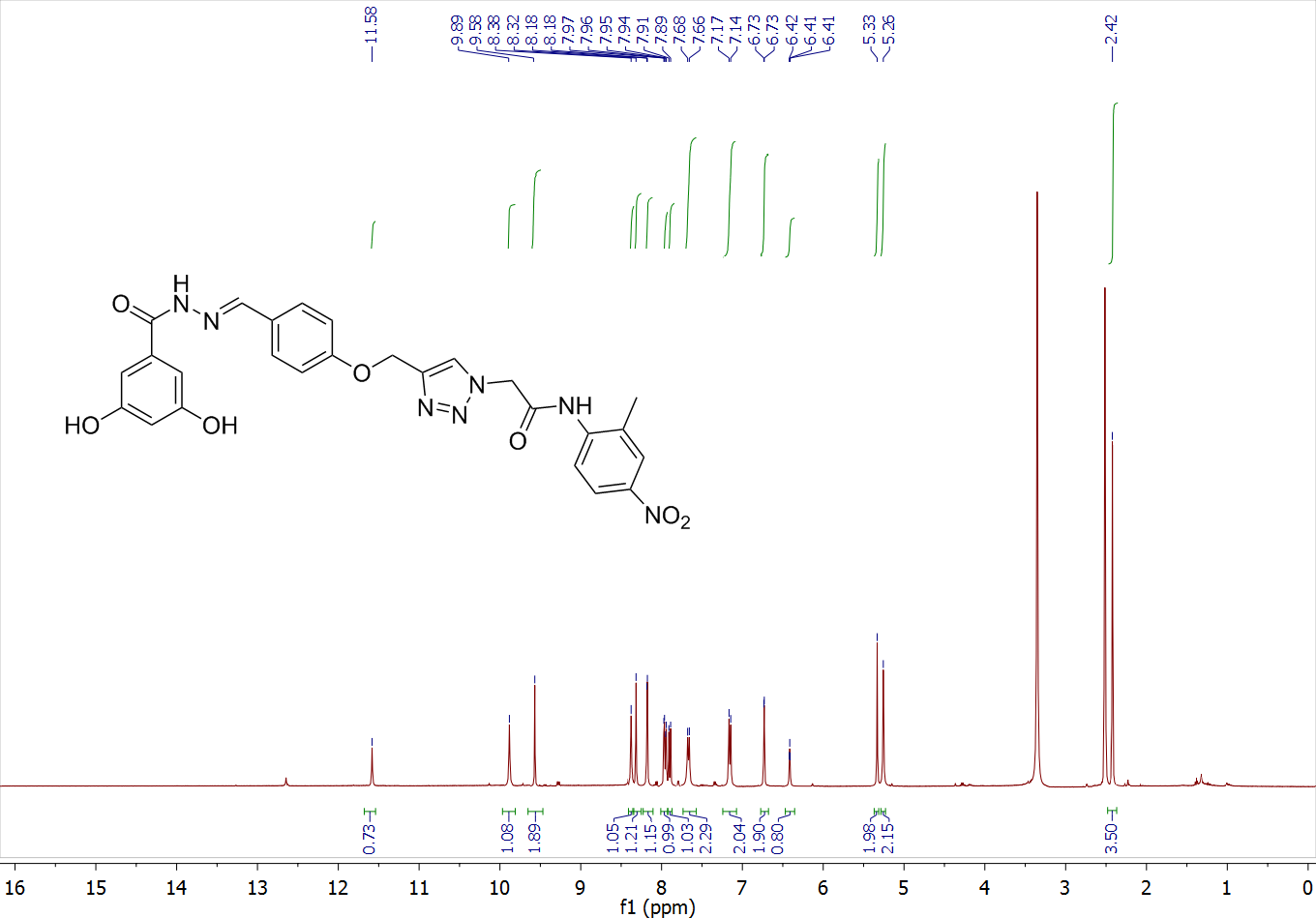


**Fig. S17.** ^1^H-NMR of **11i**


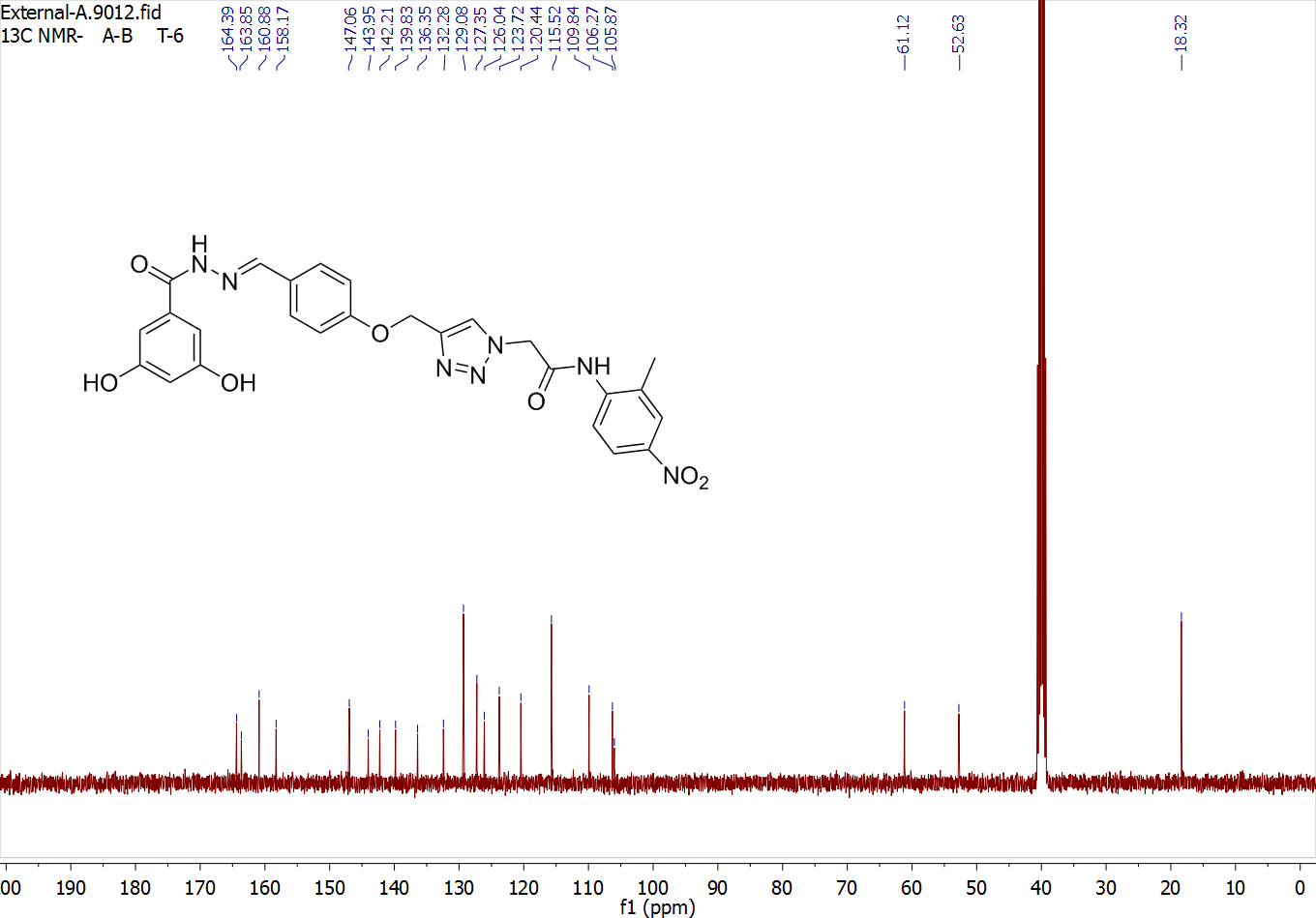


**Fig. S18.** ^1^H-NMR of **11i**


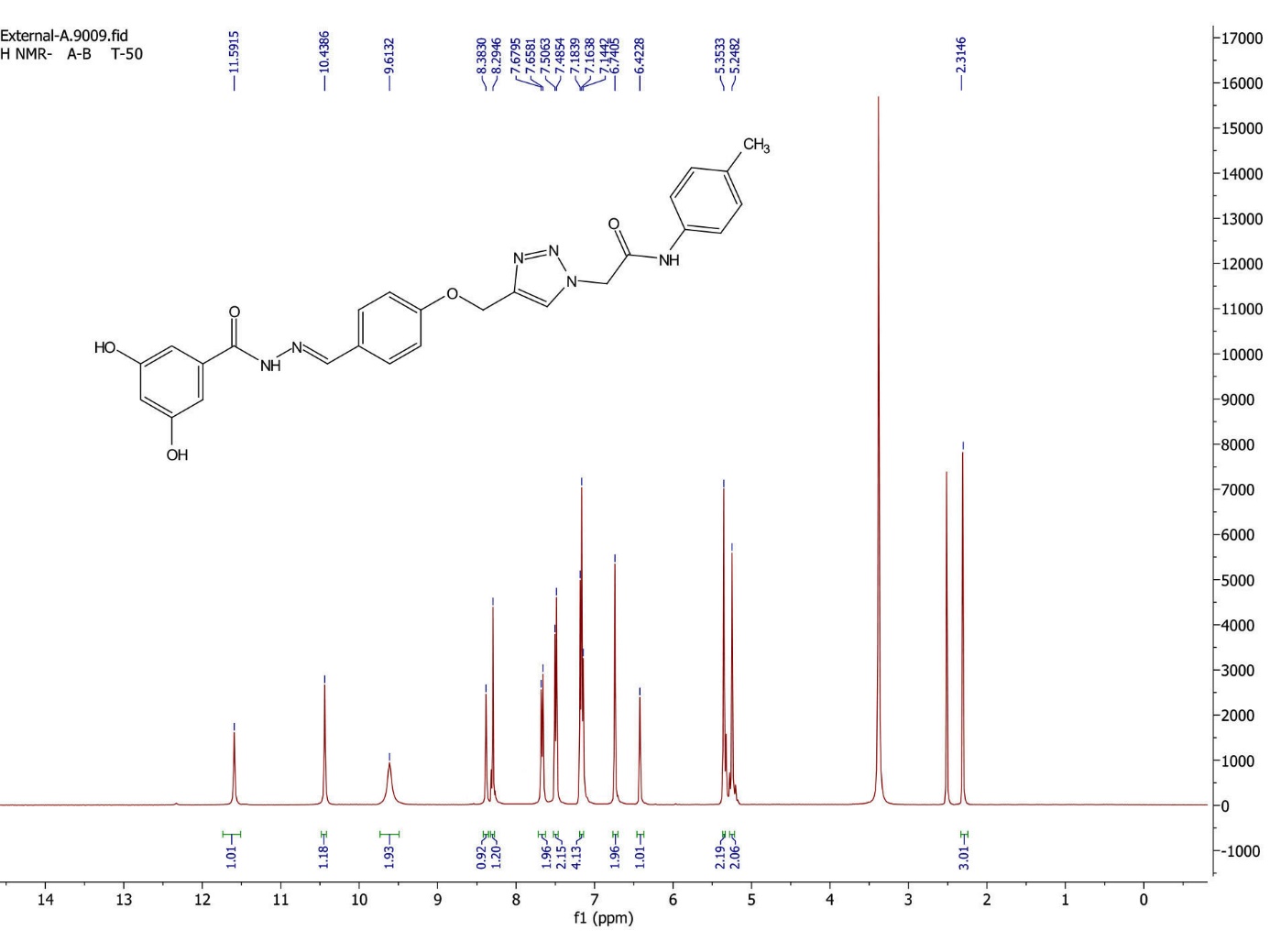


**Fig. S19.** ^1^H-NMR of **11j**


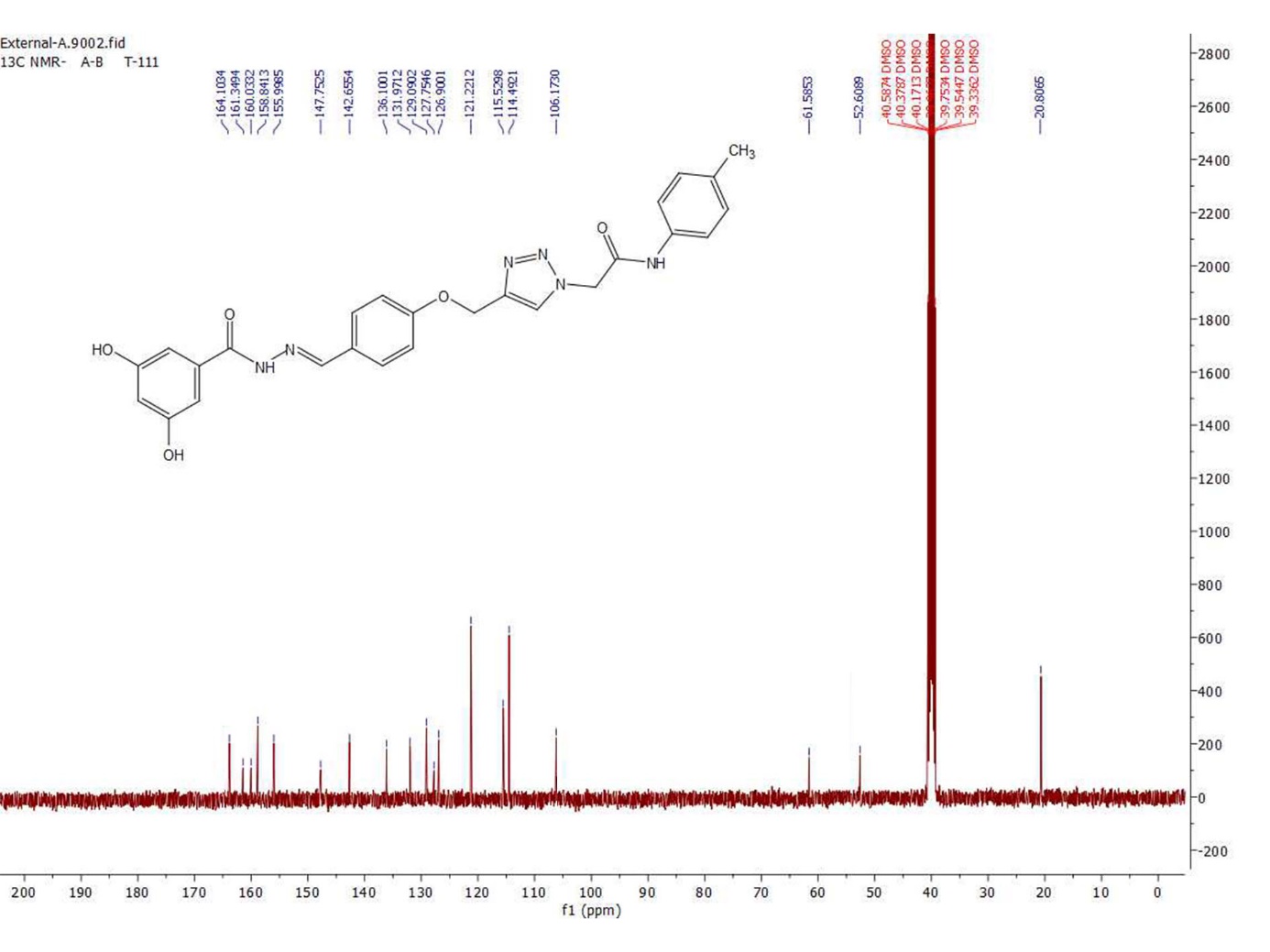


**Fig. S20.** ^13^C-NMR of **11j**


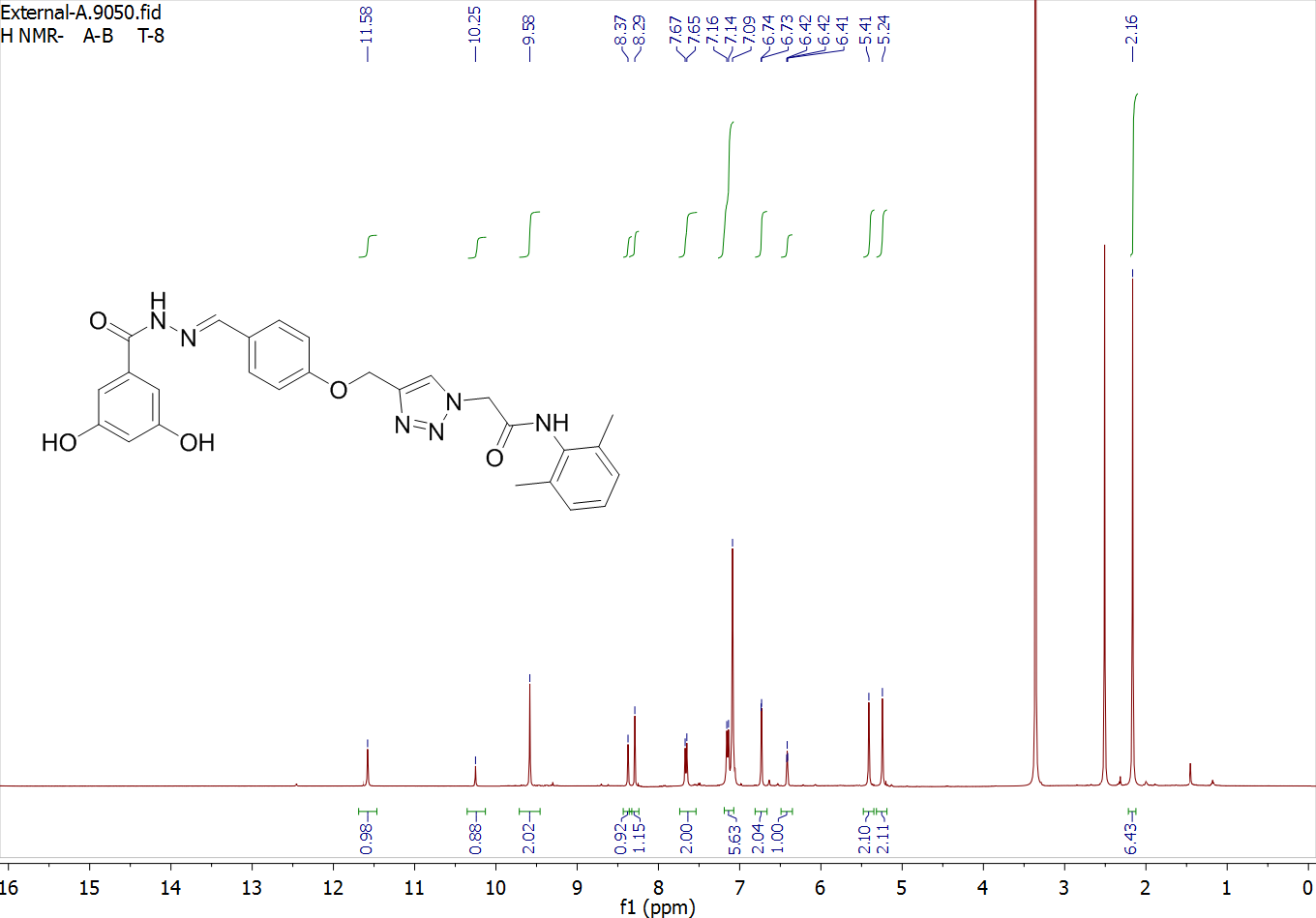


**Fig. S21.** ^1^H-NMR of **11k**


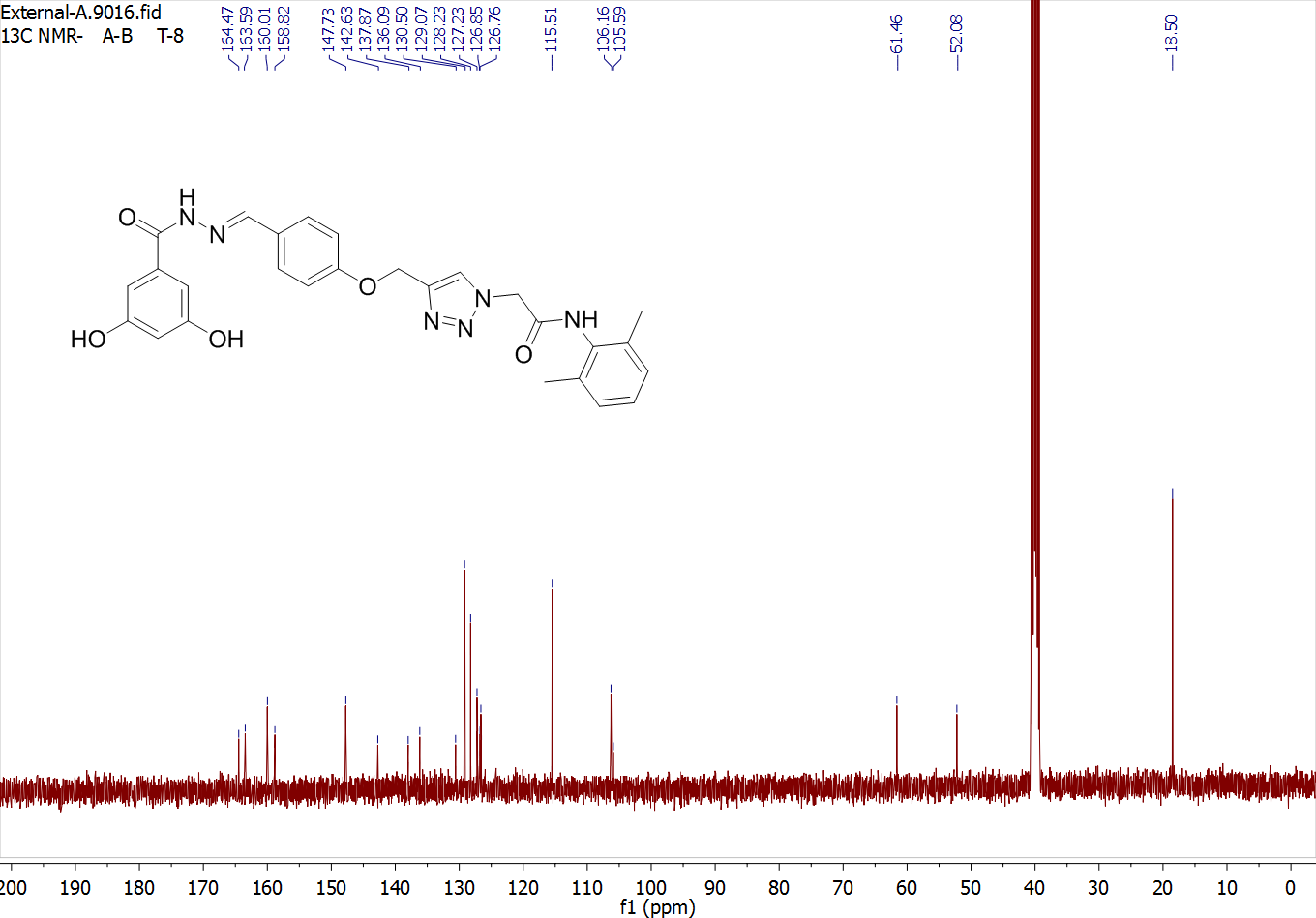


**Fig. S22.** ^13^C-NMR of **11k**


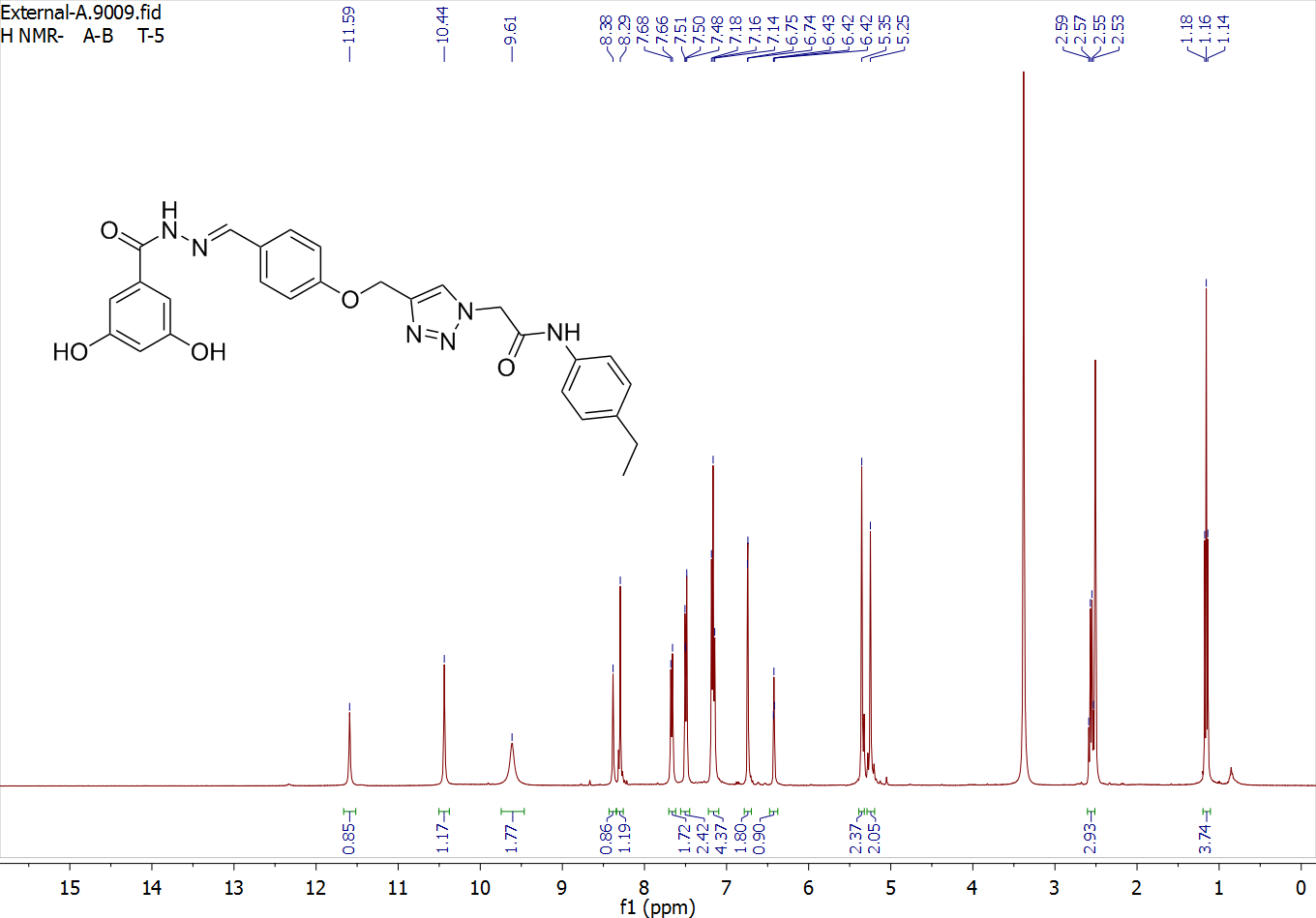


**Fig. S23.** ^1^H-NMR of **11l**


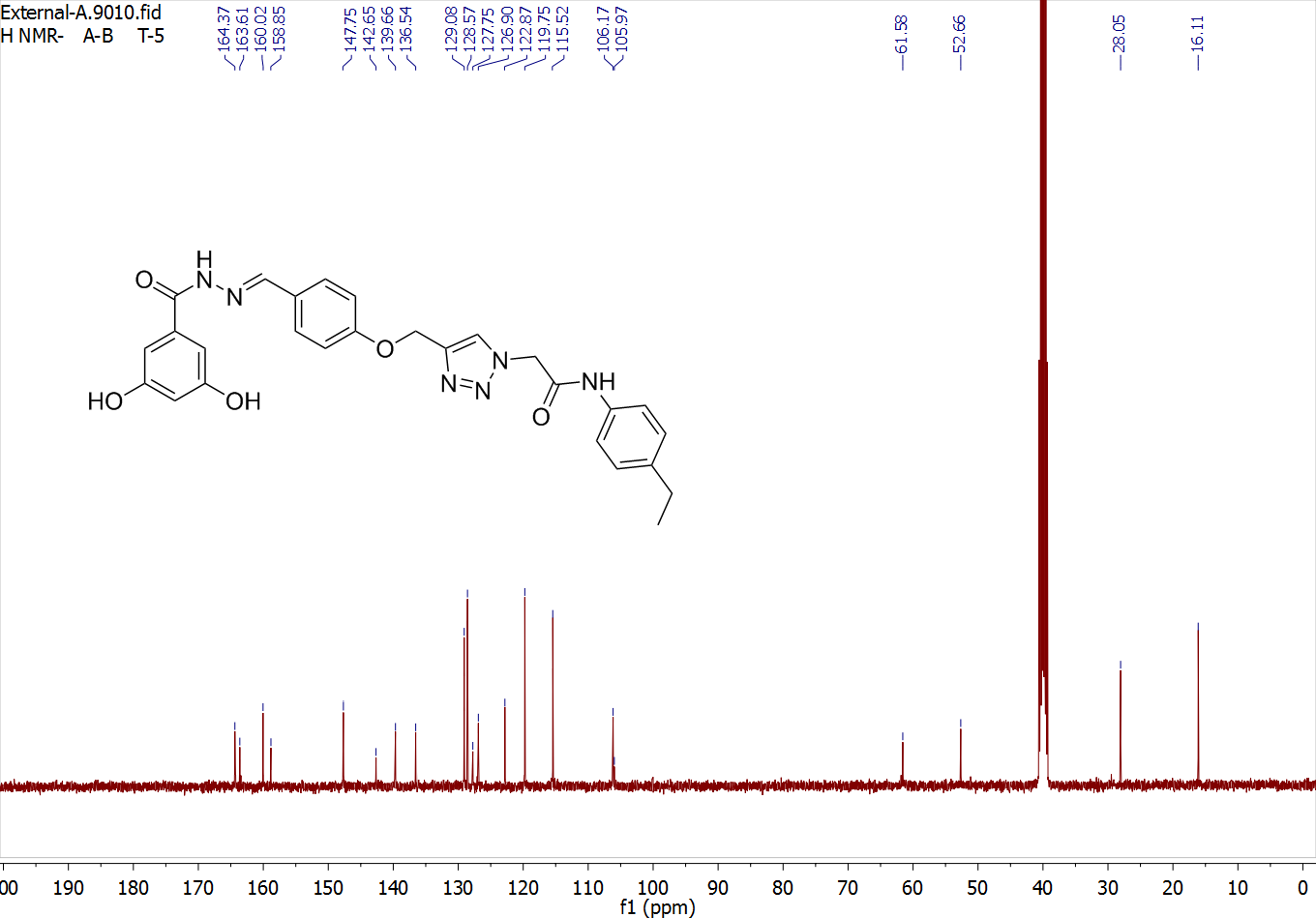


**Fig. S24.** ^13^C-NMR of **11l**


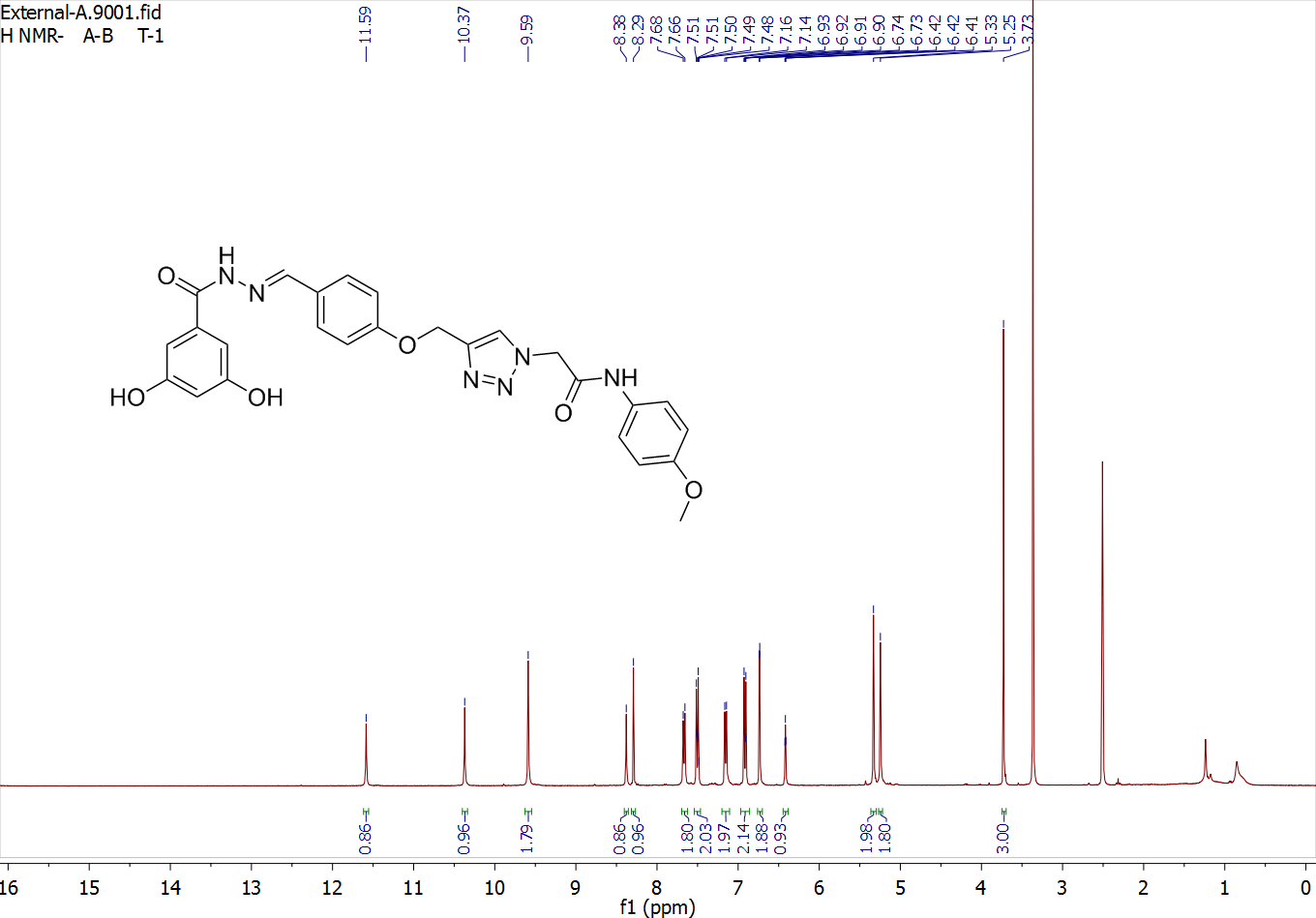


**Fig. S25.** ^1^H-NMR of **11m**


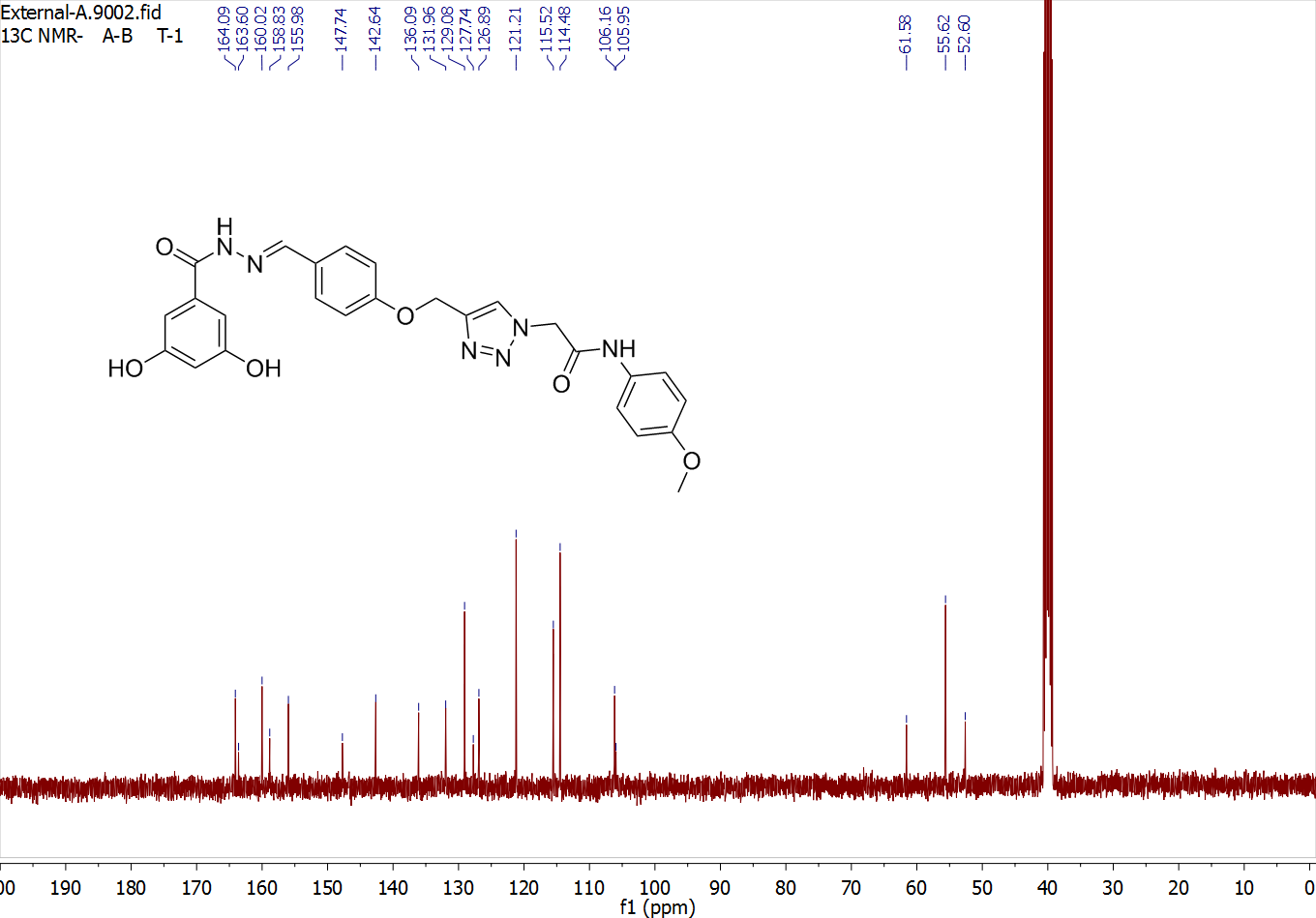


**Fig. S26.** ^13^C-NMR of **11m**


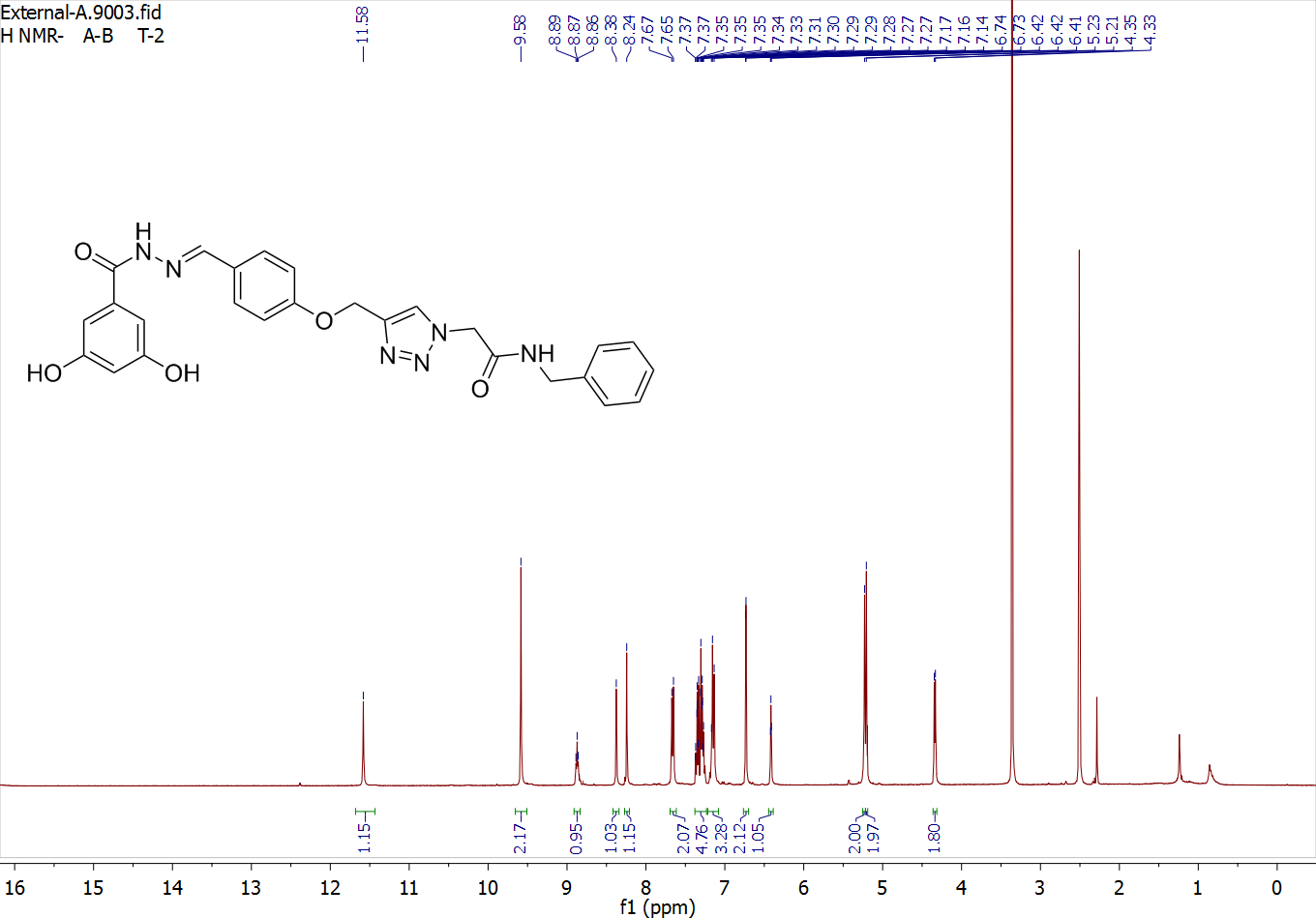


**Fig. S27.** ^1^H-NMR of **11n**


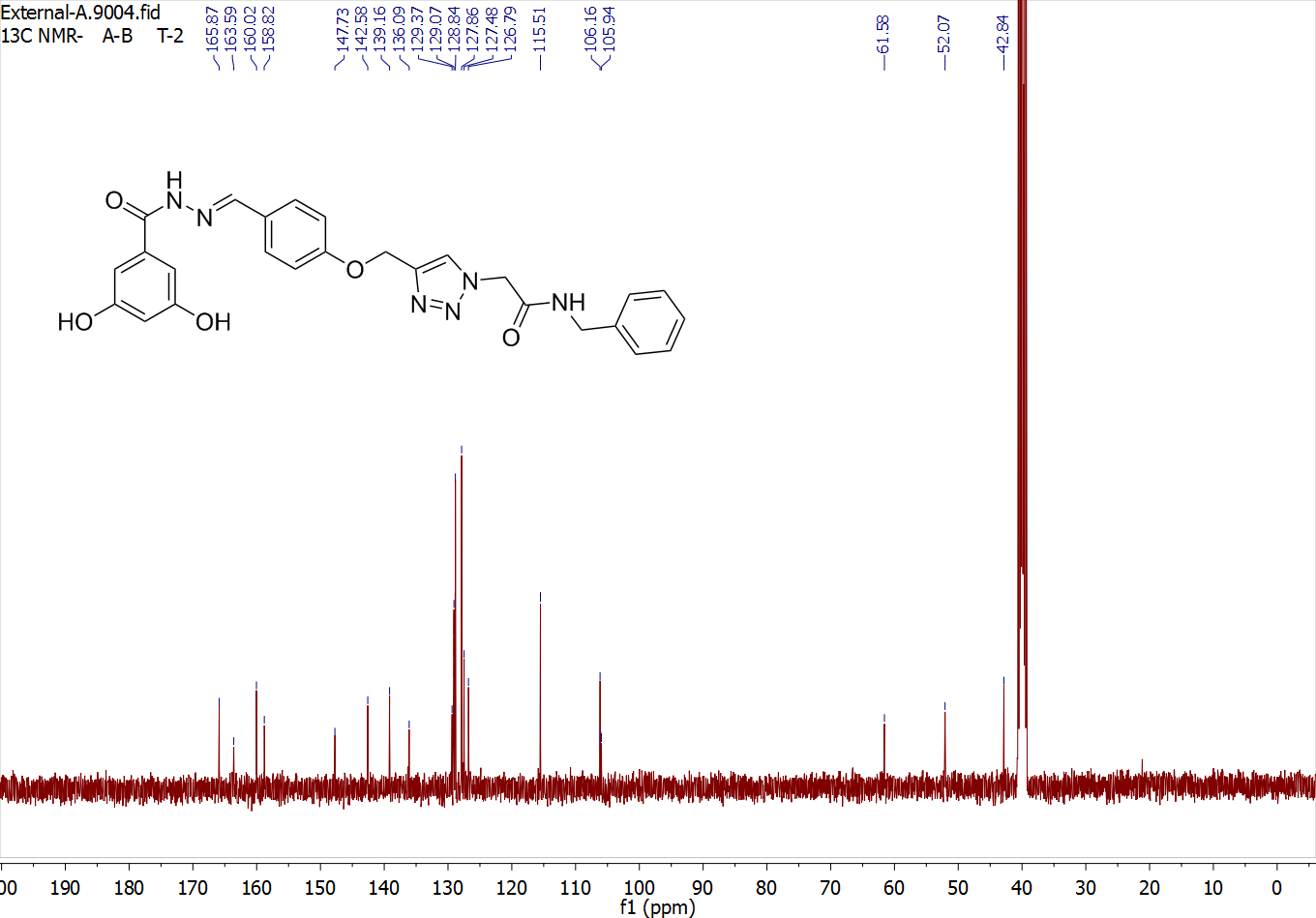


**Fig. S28.** ^13^C-NMR of **11m**
